# Differentiable rotamer sampling with molecular force fields

**DOI:** 10.1093/bib/bbad456

**Published:** 2023-12-12

**Authors:** Congzhou M Sha, Jian Wang, Nikolay V Dokholyan

**Affiliations:** Department of Engineering Science and Mechanics, Penn State University, University Park, PA USA; Department of Pharmacology, Penn State College of Medicine, Hershey, PA USA; Department of Pharmacology, Penn State College of Medicine, Hershey, PA USA; Department of Engineering Science and Mechanics, Penn State University, University Park, PA USA; Department of Pharmacology, Penn State College of Medicine, Hershey, PA USA; Department of Biochemistry and Molecular Biology, Penn State College of Medicine, Hershey, PA USA; Department of Chemistry, Penn State University, University Park, PA USA; Department of Biomedical Engineering, Penn State University, University Park, PA USA

**Keywords:** molecular dynamics, Boltzmann generator, rotameric sampling, differentiable programming, neural network, statistical mechanics

## Abstract

Molecular dynamics (MD) is the primary computational method by which modern structural biology explores macromolecule structure and function. Boltzmann generators have been proposed as an alternative to MD, by replacing the integration of molecular systems over time with the training of generative neural networks. This neural network approach to MD enables convergence to thermodynamic equilibrium faster than traditional MD; however, critical gaps in the theory and computational feasibility of Boltzmann generators significantly reduce their usability. Here, we develop a mathematical foundation to overcome these barriers; we demonstrate that the Boltzmann generator approach is sufficiently rapid to replace traditional MD for complex macromolecules, such as proteins in specific applications, and we provide a comprehensive toolkit for the exploration of molecular energy landscapes with neural networks.

## INTRODUCTION

Statistical mechanics describes the behavior of large numbers of physically identical systems [1]. Molecular dynamics (MD) is the computational application of statistical mechanics to molecular systems such as proteins, nucleic acids and lipid membranes [2–5]. The fundamental postulate of statistical mechanics is that every energetically accessible microstate of the physical system is equally probable; a microstate is a partition of the total energy of the physical system to each coordinate of its Hamiltonian [1]. When many identical copies of the physical system are present such as in molecular systems at equilibrium, experimental observations reflect the overall probability distribution of microstates.

The goal of MD is to computationally sample enough microstates of a system of molecules to approximate the distribution of microstates in a biological system at equilibrium, in which there may be on the order of Avogadro’s number (${N}_A\sim{10}^{23}$) molecules. For MD, statistical equilibrium is defined as the NPT ensemble [6], in which the number of particles, pressure and temperature are fixed, and the underlying microstate probability distribution is the Boltzmann distribution for the enthalpy.

Traditional MD attempts to sample microstates by integrating Newton’s second law according to empirically determined molecular force fields [2, 3]. The underlying major assumption is that the MD trajectory is ‘ergodic’ [6], that is, given enough time steps, the trajectory will visit all microstates with a frequency given by the Boltzmann distribution. However, there is no guarantee that a given MD trajectory will be ergodic. Transitioning between states that are separated by large energy barriers presents a significant challenge for MD simulations [7]. Numerous approaches have been proposed to address this shortcoming of MD, such as Monte Carlo methods [8–11], metadynamics [12] and umbrella sampling [13]. Recently, Boltzmann generators (BGs) have emerged as a promising candidate for replacing MD [14, 15].

Foundational methods were proposed by Noé *et al*. [14] to use generative neural networks for the sampling of microstates. The central idea is that instead of predicting a single trajectory as in MD, one may instead train a neural network to predict Boltzmann-distributed states. This approach seeks to train a neural network to learn multiple energy minima simultaneously. While Noé *et al*. successfully demonstrated their method for simple physical systems and small proteins, there were critical theoretical and practical deficiencies limiting the application of their methods that we address in this work.

The theoretical deficiencies in the original BG framework we address are various biases in angle generation due to (i) the use of a Gaussian ansatz for molecular degrees of freedom and (ii) the regularization of a discontinuous output. The practical deficiencies we address are (iii) tight coupling between energy and entropy estimation, necessitating millions of evaluations of an external molecular force field, (iv) potential numerical instabilities due to reliance on eigendecomposition and (v) inefficiencies in the generation of rotamers. We will describe how we address these five deficiencies in the results, with detailed and technical discussions relegated to the Methods.

In this work, we demonstrate that decoupling the energy and entropy training losses and propagating forces directly from the molecular force field reduces the needed evaluations of the force field by a factor of a thousand; we achieve sampling comparable to traditional MD with only ~10 [3] evaluations of the force field for chicken villin headpiece (PDB ID 1VII), a 35-residue protein domain. We demonstrate a simple method of gradient propagation for an arbitrary external force field, and we implement the AMBER 14 force field [2] in pure PyTorch [16], as is done in the TorchMD framework [17]. We include the Generalized Born implicit solvent [18–22], which is not present in TorchMD [17]. We suggest strategies to avoid numerical instabilities and intrinsic biases in the neural network, and we propose a code-efficient method of rotamer sampling that accommodates arbitrary molecules while remaining end-to-end differentiable for neural network training. We also present a highly parallel and memory-efficient version of the rotamer sampling algorithm. The result of these improvements is a numerically robust and fast architecture for BGs.

## METHODS

### Theoretical considerations for Gaussian versus non-Gaussian inputs to Boltzmann generators

One major theoretical limitation of traditional MD that carries over to BGs is difficulty in sampling disconnected local energy minima (i.e. metastable states). Fundamentally, the neural networks used in BGs are differentiable models that generate $n$ molecular internal coordinates from $m$ latent variables and are therefore continuous functions from ${\mathbb{R}}^m$ to ${\mathbb{R}}^n$. The BGs originally proposed by Noé *et al*. [14] generate internal coordinates from the sampling of a single multidimensional Gaussian distribution


(1) \begin{equation*} z\in{\mathbb{R}}^m\sim N\left(\mu =\mathbf{0},\Sigma =\boldsymbol{I}\right) \end{equation*}

centered at $\mu =0$ with unit standard deviation in each coordinate. In Noé *et al*., $m$ and $n$ were both set to three times the number of atoms in the protein (i.e. the 3D coordinates). This distribution is spherically symmetric; however, in high dimensions, the volume of the unit $m$-ball tends to 0 even for modest values of $m$, implying that the density of the multidimensional Gaussian distribution is highly concentrated near the origin. Meanwhile, the probability density of the molecule’s Boltzmann distribution is highly concentrated in disjoint regions of ${\mathbb{R}}^n$ since the energy minima of a molecule are separated by high energy barriers (low Boltzmann probability). Since $z$ tends to the origin in the latent space, we are asking the neural network to approximate a one-to-many relation, which is not a function, let alone a continuous one. Instead, it may be beneficial to sample $z$ from a sum of Gaussians


(2) \begin{equation*} z\sim{\sum}_iN\left(\mu ={\boldsymbol{x}}_i,\Sigma =\boldsymbol{I}\right) \end{equation*}

and to require that the result of sampling from distinct regions ${x}_i\ne{x}_j$ results in internal coordinates ${p}_i$ and ${p}_j$ that are also disjoint, such as through a repulsive loss on those pairs of ${p}_i,{p}_j$. This method would be analogous to metadynamics sampling [12] in which previously generated molecular states are avoided and formulated in the distributional sense. This method is also analogous to *k*-means clustering in which each centroid is responsible for representing a single cluster in the data. Alternatively, one could use an ensemble of neural networks, with each neural network responsible for generating Boltzmann-distributed states for a single energy minimum and its neighborhood of conformations.

### Differentiable force fields

Using OpenMM 7.7 [23] as our reference, we rewrote force field terms from OpenMM in terms of pure PyTorch operations, allowing for automatic differentiation of the molecular energy without the memory overhead of repeatedly transferring positional data between PyTorch [16] and OpenMM. Our implementation creates a custom PyTorch function for a provided molecule, which stores the computational graph necessary to reproduce its energy.

If such an implementation is not available for a custom energy function, one may refer to the Supplemental Methods (*Ad Hoc* Propagation of Forces, Physical Interpretation of *U*_new_) and Algorithm S1: FFDiff.

A comprehensive and technical accounting of our methods is found in the Supplemental Methods (Rewriting Molecular Force Fields to be End-to-End Differentiable, Avoiding Singularities in the Energy Function During Backpropagation). We verified that energies and gradients from our PyTorch implementation of the AMBER force field matched that of OpenMM to within 5% (Data and Materials Availability).

### Preparation of macromolecule graph metadata for rotameric sampling

Given an arbitrary macromolecule whose atoms are covalently bonded into a single connected structure, we sought to modify only the dihedral angles.

First, we created an undirected graph [24] $G=\left(V,E\right)$ of the macromolecule, with atoms as the nodes $V$ and covalent bonds as the edges $E\subseteq V\times V$.

Second, many macromolecules contain cycles that reduce the number of degrees of freedom by one, so we performed a depth-first search (which generates a tree) to break cycles at a single bond and retaining all other bonds as dihedral degrees of freedom: $T=\left(V,{E}_T\right)$.

Third, we removed all leaf nodes and edges terminating on leaves since the bonds corresponding to such edges do not represent a dihedral angle: ${T}_{dih}=({V}_{dih},{E}_{dih}$). For each remaining edge $e\in{E}_{dih}$, we assigned an output of the neural network to control the dihedral angle for that bond.

Finally, for each dihedral edge $e\in{E}_{dih}$, we used depth-first search to calculate the two connected components of ${T}_{dih}$ that result with the removal of $e$. We recorded the smaller of the two connected components, $CC(e)\subseteq V$, as the atoms which we would rotate about the dihedral axis represented by the $e$.

This method is summarized in Algorithm S2: PrepRot.

### Differentiable rotamer sampling

We started with the $N\times 3$ matrix of positions ${x}_{ij}^{(0)}$ ($1\le i\le N,1\le j\le 3$) of all the atoms in the macromolecule. For each of the angles ${\theta}_m$ predicted by the neural network, we selected the corresponding edge ${e}_m=\left({u}_m,{v}_m\right)$, where ${u}_m$ and ${v}_m$ represent the two atoms forming the covalent bond. We calculated the axis of rotation with components


(3) \begin{equation*} {k}_m={p}_{u_m}-{p}_{v_m} \end{equation*}

using the positions of atoms ${u}_m$ and ${v}_m$, which have components ${\left({p}_{u_m}\right)}_j={x}_{u_mj}^{(m)}$ and ${\left({p}_{v_m}\right)}_j={x}_{v_mj}^{(m)}$. We did not prefer a specific orientation for each rotation axis since we predicted the full $2\pi$ range for each ${\theta}_m$. We also computed the centroid of the bond as the origin for rotation:


(4) \begin{equation*} {c}_m=\frac{1}{2}\left({p}_{u_m}+{p}_{v_m}\right) \end{equation*}

We normalized ${k}_m$ to a unit vector, ${\hat{k}}_m$. We used Rodrigues’ rotation formula [25] for rotation about an axis by an angle, thereby computing a rotation matrix $R\left({\theta}_m,{\hat{k}}_m\right)$:


(5) \begin{equation*} R\left({\theta}_m,{\hat{k}}_m\right)=I+\left(\sin{\theta}_m\right){K}_m+\left(1-\cos{\theta}_m\right){K}_m^2 \end{equation*}

(6) \begin{equation*} {K}_m=\left[\begin{array}{@{}ccc@{}}0& -{k}_z& {k}_y\\{}{k}_z& 0& -{k}_x\\{}-{k}_y& {k}_x& 0\end{array}\right] \end{equation*}

We then took the previously calculated connected component $CC\left({e}_m\right)$, translated all the particles in that connected component by $-{c}_m$, applied the rotation matrix ${R}_m$ and translated the selected particles back by ${c}_m$:


(7) \begin{equation*} {x}_{ij}^{\left(m+1\right)}=\left\{\begin{array}{@{}c}{\left(\left[{R}_m\cdotp \left({p}_i-{c}_m\right)\right]+{c}_m\right)}_j,\mathrm{if}\ i\in CC\left({e}_m\right)\\{}{x}_{ij}^{(m)},\quad \mathrm{otherwise}\end{array}\right. \end{equation*}

After performing this transformation for the $M$ dihedrals we wish to sample, we return our final position matrix as the output, ${x}_{ij}^{\left(M+1\right)}$.

Our method of differentiable rotamer sampling is summarized in Algorithm S3: DiffRot.

### Alignment of point clouds

There were two methods we could have used to align point clouds. The first was the well-known Kabsch algorithm [26], and the second was through an alternative approach using unit quaternions [27, 28]. We chose to employ the second method since it reduced to a largest magnitude eigenvalue/eigenvector problem. The mathematical details for this method are found in the Supplemental Methods (The Kabsch Algorithm and A Quaternionic Alternative).

The quaternion approach is summarized in Algorithm S4: QuaternionAlignment.

### Parallelization of differentiable rotamer sampling

To take advantage of the parallel computing potential of GPU and TPU backends, we followed an approach like that of AlQuraishi’s parallelized natural extension reference frame (pNeRF) algorithm [29, 30]; however, our method generalizes to all rotational degrees of freedom. The algorithm is described in detail in the Supplemental Methods (Technical Details of Parallelized Differentiable Rotamer Sampling). We also discuss the computational advantage of this method in the Supplemental Methods (Memory Advantage of Parallelizing the Differentiable Rotamer Sampling Method).

Our parallelized version of differentiable rotamer sampling is summarized in Algorithm S6: DiffRotParallel. For practical purposes, lines 1–21 in Algorithm S6 only need to be executed once, and the rotamer sampling from lines 22 to the end may be placed in a separate function.

### Bias-free, continuous representation of dihedral angles

We used simple feedforward neural networks, with a continuous representation of angles for the output [31]. To avoid biasing predicted angles, we did not predict angles directly. For each dihedral angle ${\theta}_m$, we used our neural network to predict two parameters, $\left({x}_m,{y}_m\right)$, and calculated ${\theta}_m=\mathrm{atan}2\left({y}_m,{x}_m\right)$. $\mathrm{atan}2$ and its derivative are well defined for all $\left({x}_m,{y}_m\right)$ in all four quadrants and produces an angle in the range $\left[-\pi, \pi \right)$. To regularize our neural networks and prevent $\left({x}_m,{y}_m\right)$ from drifting to the origin or infinity, we added a loss to our training cost, with weight ${\epsilon}_{xy}$:


(8) \begin{equation*} {L}_{xy}={\epsilon}_{xy}\sum_m{\left({x}_m^2+{y}_m^2-1\right)}^2 \end{equation*}

This training cost has the advantage of being rotationally symmetric so that there is no preferred angle. For comparison, the regularization loss


(9) \begin{equation*} {L}_{\theta }={\epsilon}_{\theta}\left(\sum_{\theta_m<{\theta}_{\mathrm{min}}}{\theta}_m^2+\sum_{\theta_m>{\theta}_{\mathrm{max}}}{\theta}_m^2\right) \end{equation*}

will bias every angle ${\theta}_m$ toward $\frac{1}{2}\left({\theta}_{\mathrm{max}}+{\theta}_{\mathrm{min}}\right)$ since the network is penalized for exploring the space near ${\theta}_{\mathrm{max}}$ and ${\theta}_{\mathrm{min}}$^14^. In simple terms, the penalization of $\theta <{\theta}_{\mathrm{min}}$ and $\theta >{\theta}_{\mathrm{max}}$ results in a bleed-through effect on ${\theta}_{\mathrm{min}}<\theta <{\theta}_{\mathrm{max}}$, especially near the limits. We demonstrate this bias through a simple Markov chain [32] model of training in the Supplemental Methods (A Simplified Demonstration of Biased Sampling due to a Discontinuous Mapping).

### Estimation of entropy

For estimation of distribution entropy of our Boltzmann generators, we used a method tailored for multivariate circular distributions [33], which attempts to mitigate correlations among the angles. The metric for two sets of angular samples $\phi, \psi$ was defined as the arclength on the unit circle for each angle


(10) \begin{equation*} {d}^2\left(\phi, \psi \right)=\sum_i{\left[\pi -\left|\pi -\left|{\phi}_i-{\psi}_i\right|\right|\right]}^2 \end{equation*}

Given a batch of samples $\Phi =\left[\phi, \psi, \cdots, \omega \right]$, we then computed the nearest neighbor for each sample in the batch ${\Phi}^{\prime }=\left[{\phi}^{\prime },{\psi}^{\prime },\cdots, {\omega}^{\prime}\right]$ according to the metric $d$, and estimated the entropy of each sample as (Eq. 17 in the original manuscript [33], with first nearest neighbors corresponding to $m=1$)


(11) \begin{equation*} {H}_{\phi }=\log d\left(\phi, {\phi}^{\prime}\right) \end{equation*}

and averaged over the entire set of samples $\phi \in \Phi$. Since we were only using the entropy to provide approximate gradients to the Boltzmann generator, we ignored all constants that corrected for bias to the numerical value of the entropy in the original formula.

## RESULTS

We begin with an overview of the changes we made to the BG framework to address the five issues enumerated in the introduction. We first address the theoretical concerns (i) and (ii), then we discuss aspects of our implementations addressing practical concerns (iii)–(v), and finally we discuss the results and basic benchmarking of our proposed methods.

### (i) A Gaussian ansatz for the latent space is biased toward the origin

BGs are continuous functions between a latent space of random variables and a target space representing physical configurations, so we will denote elements of the latent space by $x$, the BG by $f$ and the generated physical configurations by $y=f(x)$.

The Kullback–Leibler (KL) divergence [34] measures differences between the generated $f(x)$ and the true Boltzmann distribution $p(x)$. BGs are therefore trained to minimize the KL divergence, to reproduce the physical configurations expected from statistical mechanics [14]. The original BG framework constrains the functional form of $f$ in a variety of ways to ensure that the estimate of the KL divergence is directly computable, with fixed formulas for all the terms involved, such as $f$, ${f}^{-1}$ and $\log J$, where $J$ is the Jacobian determinant of $f$. The first constraint on the functional form of $f$ is that the input for $f$ is a vector of Gaussian random variables.

In molecular systems, there are often multiple metastable/low-energy states, which are represented by high-probability islands of the target space which are separated by a sea of low-probability states. We therefore face a fundamental difficulty, if we seek to find functions $f$ that map a Gaussian vector to such a target space. In high dimensions, Gaussian vectors are highly concentrated in a single location: the origin. This can be seen through the volume of a unit ball [35], which is exponentially suppressed with the dimension, $V\sim{e}^{-d}$. Since Gaussian vectors are spherically symmetric, the proportion of configurations they represent as compared to the volume of the entire target space also falls exponentially. This implies that the Jacobian determinant of $f$ must be exponentially large with the dimension so that the generated physical configurations are not stuck near a single state, which in practice leads to numerical instability. For large molecular systems like proteins, even a coarse-grained model rapidly leads to a large value of $d$ since we must predict many degrees of freedom, thus leading to high suppression of $f$. Additional discussion may be found in the Methods (Theoretical Considerations for Gaussian Versus Non-Gaussian Inputs to Boltzmann Generators).

### (ii) Naïve angle sampling by transforming a single variable is discontinuous

In the original BG framework, angles were generated using a single random variable, and the BG was trained to produce values in $\left[0,2\pi \right)$ for each angle, for example for a backbone dihedral angle in a protein. The physical reality is that angles should be $2\pi$ periodic, that they fundamentally lie on circles. Since the BG is a continuous function, we, therefore, require that they map the latent space to the target space continuously; the transformation introduced in the original BG framework is discontinuous because it seeks to map a line segment to the circle [31].

Even ignoring this fundamental difficulty, the original BG framework trained the network to produce angles by penalizing the network for producing outside of $\left[0,2\pi \right)$. This method is biased, even for angles within that range. By penalizing angles produced outside of the range, the original BG framework penalized angles close to the limits by making it difficult for the network to produce angles close to 0 and $2\pi$, intuitively because producing angles too close to the limits leads to a greater likelihood that the network will be penalized in the next training step. In other words, the network is in danger of producing invalid outputs if we allow it to stray too close to the boundary. We demonstrate this bias against producing angles close to the boundary in the Supplemental Methods (A Simplified Demonstration of Biased Sampling due to a Discontinuous Mapping). Using a simple, discrete Markov chain model of training, we explicitly show that when the network is fully trained (i.e. the training process is at equilibrium), the probability of the network producing a given angle is reduced near the boundaries relative to the center of the interval.

### Resolving (i) and (ii) using bias-free, continuous representation of angles

We show in the Methods (Bias-Free, Continuous Representation of Dihedral Angles) how to resolve both (i) and (ii), a mathematical prescription introduced specifically for 2D angle generation [31]. In the original BG framework, each angle $\theta$ corresponds to a single output of $f(x)$. In the bias-free continuous method, each angle $\theta$ is instead represented by two outputs, $c=f(x)$ and $s=g(y)$, where we have suggestively labeled the generated numbers $c$ and $s$ to imply $\cos \theta$ and $\sin \theta$. We then calculate


(12) \begin{equation*} \theta =\mathrm{atan}2\left(s,c\right) \end{equation*}

If we assume that $x$ and $y$ are drawn from distributions centered at $0$, and $f$ and $g$ are similarly initially unbiased, then $\theta$ is also drawn bias-free from $\left[0,2\pi \right)$, and training of $f$ and $g$ proceeds in an unbiased fashion, with no difficulties near the interval boundaries. Furthermore, the gradient of $\mathrm{atan}2$ is well defined and bounded:


(13) \begin{equation*} \nabla\ \mathrm{atan}2\left(s,c\right)=\frac{1}{c^2+{s}^2}\left(-s,c\right), \end{equation*}

and therefore backpropagation through $\mathrm{atan}2$ does not introduce an exploding gradient problem.

Finally, the $n$ rotameric degrees of freedom of a molecule encompass the space ${\left[0,2\pi \right)}^n$. Since $c=f(x)$ and $s=g(y)$ are assumed to be rotationally symmetric initially, the resulting angles from Eq. (52) represent the uniform distribution on ${\left[0,2\pi \right)}^n$. Therefore, we only require the Jacobian determinants of $f$ and $g$ to be $O(1)$, and we avoid the exponential scaling problems found in the original BG framework.

**Figure 1:**
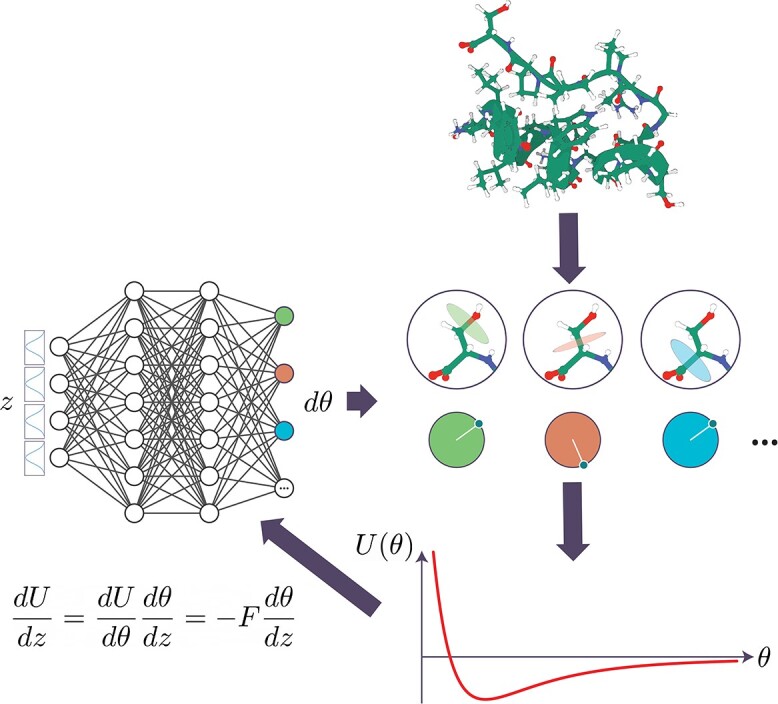
Differentiable rotamer sampling for Boltzmann generators. Given an arbitrary macromolecule, we identify all the rotameric degrees of freedom; in the case of a protein, these degrees of freedom are the dihedral angles of backbones and side chains. We use a neural network to generate changes to the dihedral angles and perform the rotations on the protein structure. Finally, we evaluate the energy and forces of the resulting structure with respect to a molecular force field, and backpropagate the gradients through the sampling process to the neural network. In this way, we can train the neural network to produce states with low energy, allowing for the study of potential stable conformations of the macromolecule.

The general architecture of our method is shown in Figure 1.

### (iii) Decoupling entropy estimation and energy function evaluation improves training speed by orders of magnitude

In the original BG framework, energy and entropy estimation are performed in a single calculation, necessitating as many calls to the energy function as calls to the neural network to generate physical configurations. The calls to the energy function are especially burdensome when the energy function is not implemented in the same framework as the neural network, causing a memory transfer bottleneck in (1) transferring generated coordinates from the neural network to the external energy function and (2) transferring energies and gradients from the external energy function to the neural network. Even when the energy function is written in the same framework as the neural network, the evaluation of a molecular force field is computationally much more expensive than coordinate generation by the neural network. We found that decoupling the energy portion of the loss function from the entropy estimation portion of the loss function resulted in many orders of magnitude faster training, with far fewer evaluations of the energy function necessary.

### (iv–v) Eigendecomposition-free fragment assembly for efficient rotamer sampling

The original BG framework used singular value decomposition to remove the translational and rotational degrees of freedom encountered during sampling. Because we sample the rotameric degrees of freedom directly, we do not need to perform such eigendecomposition, which can be numerically unstable when backpropagating gradients, as the documentation for PyTorch’s torch.linalg.svd indicates [16].

**Figure 2:**
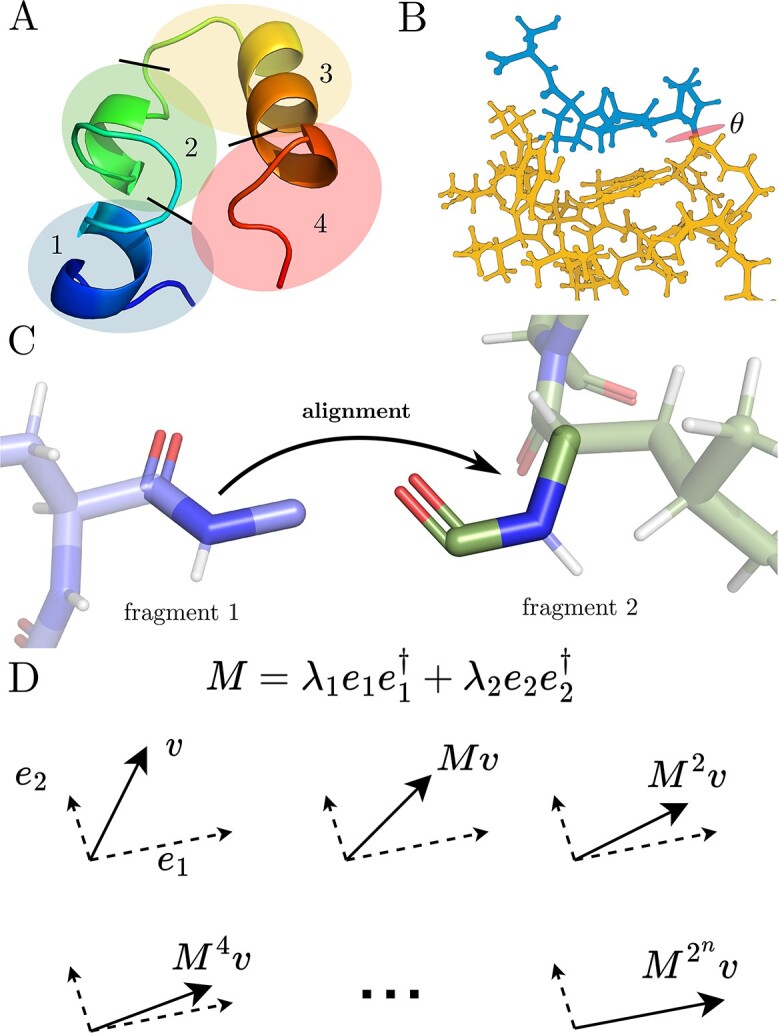
Differentiable rotamer sampling workflow. (**A**) The protein (chicken villin headpiece, PDB ID 1VII) is split into fragments, whose rotamers can be sampled in parallel. (**B**) For each rotameric degree of freedom, we split the macromolecule into two connected components at the associated bond, using a depth-first search on the graph of bonds. We then rotate (oval labeled θ) the smaller connected component (upper portion) about the bond axis by the neural network output *θ*, using Rodrigues’ formula. (**C**) To combine fragments after dihedrals have been sampled for each fragment, we use an alignment algorithm (Kabsch or quaternionic) on specific atoms in the backbone. Through this method, we can therefore explore the energy landscape of the macromolecule solely as a function of its internal, rotameric degrees of freedom. Because each step of this method is differentiable, we can backpropagate gradients through the rotamer sampling process to provide derivatives for *θ*. (**D**) Intuition for exponential acceleration of power iteration. We used repeated matrix squaring to achieve high matrix powers, leading to exponential acceleration of the traditional power iteration algorithm for finding the largest magnitude eigenvalue and associated eigenvector. With higher powers of M multiplying an arbitrary vector v, the e2 component is gradually suppressed.

**Figure 3:**
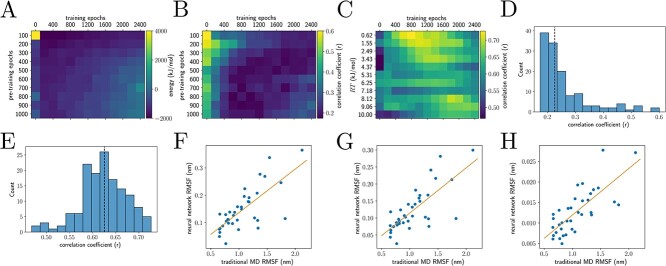
Evaluating the consequences of neural network pre-training and entropy (chicken villin headpiece, PDB ID 1VII). We only sampled the protein backbone dihedrals. (**A**, **B**) Examining the effect of pre-training to produce the native structure, as well as the role of entropy in neural network training. (A) The minimum energy structure produced from ~600 structures across 3 trained networks; not trained on the entropy. (B, **C**) Correlation coefficients between traditional MD alpha-carbon root-mean-square fluctuations (RMSF) and neural network RMSF. (B) Neural networks trained without entropic estimation; (C) neural networks trained on the full loss described in Results. Entropy training is necessary to reproduce traditional RMSFs. (**D**) Distribution of all correlation coefficients from (B), with median of 0.23 (dashed line). (**E**) Distribution of all correlation coefficients from (C), with median of 0.63 (dashed line). We performed a Fisher r-to-z transform on the data for (D) and (E) and performed a *t*-test for unequal variances (*t* = −39.78, *P* < 0.001). Training with entropy (E) better reproduces relative RMSFs than without (D). (**F**–**H**) Traditional MD RMSF versus the neural network RMSF for the four networks with the highest correlation. (F) RT = 0.62 kJ/mol, 1200 epochs, *r* = 0.73; (G) RT = 0.62 kJ/mol, 1000 epochs, *r* = 0.75; (H) RT = 9.06 kJ/mol, 200 epochs, *r* = 0.75.

**Figure 4:**
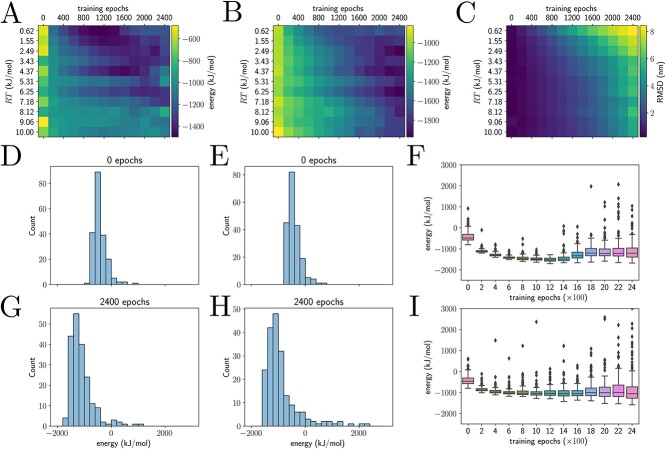
Training characteristics across a range of temperatures. In these plots, we examine the effect of training on the energies of output structures for chicken villin headpiece; for rotamer sampling, we only sampled the protein backbone dihedrals. (**A**–**C**) For each value of the temperature, we trained three neural networks, first on a loss to reproduce the native structure for 1000 epochs, and then by the Boltzmann loss of the full energy and entropy for up to 2400 epochs. Darker squares indicate lower energies, while lighter squares indicate higher energies. (A) Each square represents the median energy of ~600 generated structures across all three networks. (B) Minimum energy of the structures. (C) The median RMSD from the native structure. (**D**, **E**) Initial energy distribution at RT = 0.62 kJ/mol and 10 kJ/mol, respectively. (**G**, **H**) Final energy distribution at the same temperatures. (**F**) Energy distributions as a function of training epochs for one of the networks we trained at RT = 0.62 kJ/mol. (**I**) Energy distributions as a function of training epochs for one of the networks we trained at RT = 10 kJ/mol. Higher temperatures homogenize the sampled structures in terms of energy, in (A)–(B). In (D)–(I), we observe that training the neural networks equilibrated within a few hundred epochs of training.

**Figure 5:**
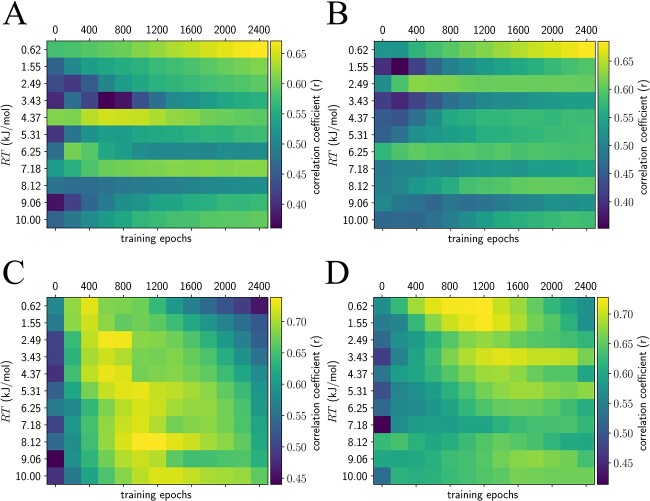
Evaluating the effect of input noise distribution and training algorithm on simulation accuracy. As before, we used chicken villin headpiece for these experiments. In (**A**) and (**B**), we used stochastic gradient descent, while in (**C**) and (**D**), we used the Adam optimizer for neural network training. In (A) and (C), we used uniform input noise while in (B) and (D) we used a sum-of-Gaussians input noise. We found that stochastic gradient descent was inferior to the Adam optimizer in reproducing RMSFs, while the choice of uniform versus sum-of-Gaussians versus single Gaussian input noise (Figure 3C) did not appear to make an empirical difference in training accuracy (maximum correlation coefficient ~0.75). As before, each entry in (A)–(D) represents the median RMSF correlation coefficient of three neural networks trained independently, evaluated across ~600 generated structures and compared to traditional MD.

In this work, we implement direct sampling of rotameric degrees of freedom in macromolecules (Figure 2), described in the Methods (Differentiable Rotamer Sampling) and associated Supplemental Methods. High-energy degrees of freedom such as bond lengths and bond angles are kept fixed, or ‘frozen out’ in the language of statistical mechanics, since they are typically inaccessible to the molecular system in its native biological environment. We demonstrate that our methods reflect traditional MD (Figures 3–5), which we will discuss more carefully in the sequel.

**Figure 6:**
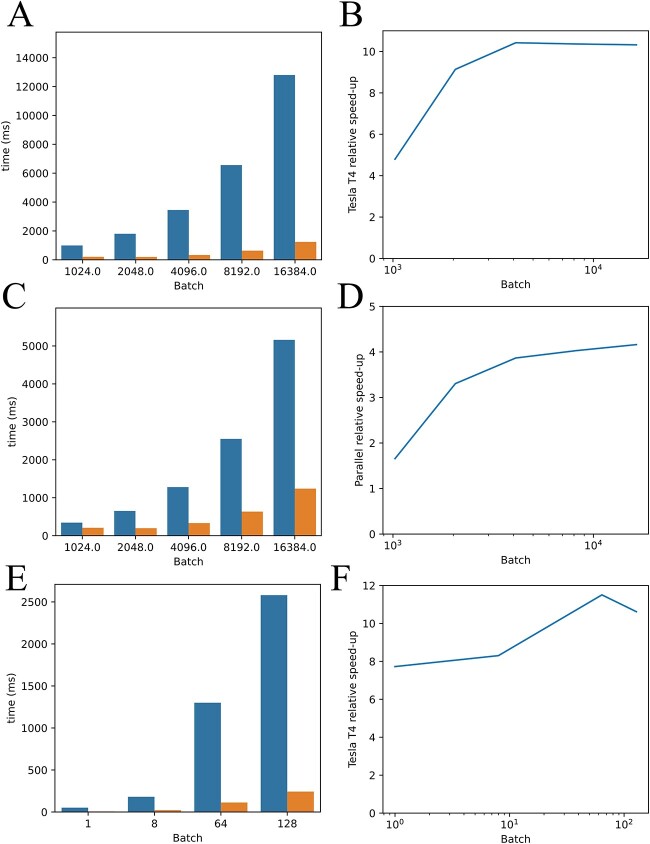
Benchmarking of rotamer sampler and the energy function written in PyTorch. (**A**, **B**) Comparing performance of the parallel dihedral sampler on chicken villin headpiece (PDB ID 1VII), split into four fragments. We compared CPU-only computation on an M1 Max MacBook Pro (left bar in each pair of bars for a given batch size) to an NVIDIA Tesla T4 GPU with 16 GB RAM (right bar). We observed a plateau above a batch size of 4096 in performance increase, indicating saturation of the GPU with CUDA kernels. (**C**, **D**) Comparing the performance of the non-parallel (blue) and parallel (orange) versions of the dihedral sampler. (**E**, **F**) Comparing the M1 Max (blue) versus the NVIDIA GPU (orange) on the energy function. For a batch size of 256 and higher, the 16 GB GPU ran out of memory.

Our method of direct sampling of rotamers can be parallelized (Methods, Parallelization of Differentiable Rotamer Sampling) by breaking up the molecule into fragments (Figure 2A), performing rotamer sampling for each fragment independently and in parallel (Figure 2B), and reassembling the fragments through 3D alignment (Figure 2C). This parallelization results in large improvements in performance, particularly with GPU acceleration (Figure 6A–D).

For assembly of the fragments, instead of relying on eigendecomposition to perform 3D alignment through the well-known Kabsch algorithm [26], we use an alternative method that relies only on finding the largest eigenvalue and associated eigenvector of a certain matrix [28], rather than finding a complete eigendecomposition (Methods, Alignment of Point Clouds). We also develop a method (Methods, Differentiable Largest Eigenvalue and Associated Eigenvector of a Square Matrix) to find the largest eigenvalue and associate eigenvector quickly and stably, which is well suited for automatic differentiation as compared to singular value decomposition (Figure 2D).

Finally, we implement the molecular force field completely in PyTorch (Figure 6E and F), avoiding the memory transfer bottleneck between the energy function and neural network components of the architecture mentioned previously.

### Basic benchmarking of differentiable rotamer sampling with molecular force fields

In the following sections, we will describe our initial evaluations of our proposed adjustments to the BG framework. See the Supplemental Methods for additional details on ‘Learning rate tuning’, ‘Neural network architectures’, ‘Traditional MD’ and ‘Order parameters’ such as RMSD and RMSF.

### Initializing Boltzmann generators at the native state

We tested the effect of pre-training the neural networks to output $\theta =0$, or in other words to produce the identity function on the structure. For these experiments (Figure 3A and B), we trained the neural network for ${n}_{\mathrm{pre}}$ epochs with the loss


(14) \begin{equation*} {L}_{\mathrm{pre}}=\sum_i{\theta}_i^2+{L}_{\mathrm{reg}} \end{equation*}

where ${L}_{\mathrm{reg}}$ includes fixed terms such as the angle modulus loss and weight decay regularization.

We observed that initial pre-training was crucial to allow neural networks to converge. With no pre-training epochs, we were unable to train neural networks within 5000 epochs, and all structures produced appeared highly unphysical, with numerically infinite energies (not shown). However, any number of pre-training epochs above 100 appeared suitable for initialization of the neural networks (Figure 3A).

### Sampling without entropy

When we trained neural networks solely on the energy and not the entropy (Figure 3A and B)


(15) \begin{equation*} {L}_{\mathrm{energy}}\left(\theta \right)=U\left(\theta \right)+{L}_{\mathrm{reg}} \end{equation*}

we observed that while the intrinsic noise in the Adam optimizer allowed for sampling of states that were not global energy minima (Figure 3A), the resulting neural networks did not reproduce the results of traditional MD at non-zero temperature (Figure 3B).

### Temperature and entropy, and effect of training length on structure generation

We observed that by estimating the multivariate circular distribution entropy and training to maximize the entropy and minimize the energy, we were able to reproduce traditional MD protein backbone root-mean-square fluctuations (Figure 3C–H), as well as prevent mode collapse (Figure 4A–C) and sample Boltzmann-distributed states (Figure 4D–I). For this set of experiments, we used the training loss


(16) \begin{equation*} {L}_{\mathrm{train}}\left(\theta; {T}_{NN}\right)=\left\{\begin{array}{@{}l}\frac{U\left(\theta \right)}{T_{NN}}-S\left(\theta \right)+{L}_{\mathrm{reg}},{T}_{NN}\ge 1\\{}U\left(\theta \right)-{T}_{NN}\cdotp S\left(\theta \right)+{L}_{\mathrm{reg}},{T}_{NN}<1\end{array}\right. \end{equation*}

where ${T}_{NN}$ is the temperature of the system (Supplemental Methods, Estimation of Temperature of Boltzmann-Generated Samples). We chose this form of the loss to maintain the relative contributions and dynamic range of the energy function $U$ and entropy $S$, while preventing exploding gradient contributions from dividing $U$ by a small ${T}_{NN}$ or multiplying $S$ by a large ${T}_{NN}$. As previously discussed, we estimated entropy independently from energy function evaluations; we used a nearest neighbor procedure tailored for measuring the entropy of multivariate circular distributions (Methods, Estimation of Entropy) [33]. In the units of our implementation,


(17) \begin{equation*} {T}_{NN}=R\cdotp T \end{equation*}

with the ideal gas constant $R=8.314\times{10}^{-3}\ \mathrm{kJ}\ {\mathrm{mol}}^{-1}\ {\mathrm{K}}^{-1}$ and physical temperature measured in Kelvins $T$. A human body temperature of $310\ \mathrm{K}$ yields a numerical value of ${T}_{NN}=2.58$. However, since our estimate of the entropy is only correct asymptotically, the actual numerical values for the temperature may differ in practice. Interestingly, it appears that reproducing the results from traditional MD is a matter of fine-tuning both the length of neural network training and temperature (Figures 3C and 4D–I).

### Training networks with non-Gaussian priors and comparing stochastic gradient descent versus the Adam optimizer

In the preceding sections, we used Gaussian noise as the initial input of our models. However, as we discussed in issue (i), multivariate Gaussians [35] may significantly limit the conformations we are able to sample (Methods, Theoretical Considerations for Gaussian Versus Non-Gaussian Inputs to Boltzmann Generators). We therefore examined for differences in RMSF accuracy based on the input noise; we also tested for differences between the Adam optimizer and stochastic gradient descent (Figure 5). We found no empirical difference among Gaussian and non-Gaussian input noises, and we found that stochastic gradient descent was inferior to Adam for reproducing RMSFs calculated by traditional MD.

### Benchmarking

We performed basic benchmarking of the rotamer sampler, the parallelized version of the rotamer sampler and the force field on the CPU-only of an M1 Max MacBook Pro with 64 GB RAM, and on an NVIDIA Tesla T4 GPU with 16 GB RAM (Figure 6). These benchmarks were performed on the combined forward and backward passes through the computational graph, to imitate real-world usage. We observed an advantage of up to 10× on the GPU in terms of total throughput of rotamer sampling (Figure 6A and B). We also compared the original non-parallel dihedral sampler (Algorithm S3) to the parallel dihedral sampling, running on the NVIDIA GPU (Figure 6C and D), which showed a performance advantage of 4× on the protein we used for these experiments. Finally, we demonstrated that the energy function also demonstrated a performance benefit running on the NVIDIA GPU compared to CPU, with 10× greater performance (Figure 6E and F).

## DISCUSSION

In this work, we addressed five categories of fundamental deficiencies in the BG framework. In deficiencies (i) and (ii), we addressed sources of neural network bias by using continuous sampling of rotameric states. Since we have addressed these theoretical issues in the Results and Methods, we will focus on the practical advantages of our proposed methods in deficiencies (iii)–(v).

### Computational throughput improvements in the BG framework

By decoupling energy minimization from entropy maximization, we were able to perform Boltzmann sampling with three orders of magnitude fewer calls to the energy function than in the original BGs. While the original BG framework required millions of evaluations of the energy function, we only required thousands to tens of thousands to reproduce traditional MD results in the form of residue-wise RMSF (Figures 3–5). Because we included the implicit generalized Born solvent, we were able to avoid the use of explicit water while simulating proteins in biologically relevant conditions.

In the original BG framework, all $3N$ atomic coordinates are predicted [14]. In contrast, we postulated that many of the degrees of freedom are unimportant to protein dynamics, and so we selectively sampled internal degrees of freedom (i.e. the dihedral angles), explicitly freezing out all other modes in the molecule. A side effect of our approach was that we did not need to manually remove modes such as overall molecular rotation/translation through eigendecomposition. Though we no longer have a closed form expression for the entropy, we gain an enormous computational advantage in the neural network.

### Model size reduction in the BG framework

A major contributing factor to our computational advantage was that our networks handle the same protein systems with far less memory and fewer trainable parameters than the original BG framework. The BG framework required that the entire neural network be invertible so that exact gradients for their KL divergence between Gaussian priors and Gaussian posteriors may be backpropagated. Thus, each layer of the original BG neural networks has the same $3N$ dimensions, otherwise the Jacobian determinant of the network would vanish and the neural networks would no longer be invertible. Even for small proteins like the chicken villin headpiece we studied, $N=596$, and thus the neural networks quickly grow in number of trainable parameters. If we restrict the number of hidden units in any layer to a value less than $3N$, then the Jacobian determinant of the neural network transformation will immediately become $0$ since the transformation is no longer full rank. Therefore, the number of parameters in an invertible network is at least $9{N}^2$ (3 196 944 for chicken villin headpiece, not including bias parameters). In practice, we require multiple hidden layers with just as many parameters to gain sufficient approximation power for the neural network, resulting in networks with tens of millions of trainable parameters, even for modestly sized proteins. Since we selectively sampled degrees of freedom that are normally not frozen out at biologically relevant energies, we were able to reduce the computational burden and memory-footprint of the neural networks used. To be concrete, we consider the example given by our results. For backbone dihedral sampling, we required only a prediction of the $\phi, \psi, \omega$ angles for each of the 36 residues in chicken villin headpiece, leading to 10-layer, fully connected models that had 196 692 trainable parameters. The equivalent model in the original BG framework would require roughly 100× as many trainable parameters.

### Memory and hardware acceleration of the BG framework

By writing both the rotamer sampler and the force field in pure PyTorch, we were able to leverage the automatic computational graph optimization, GPU acceleration and automatic differentiation features of PyTorch. By using pure PyTorch, we avoided the burden of copying data back and forth from an external energy function to PyTorch. We provide benchmarks for our algorithms, which demonstrate high throughput (Figure 6). In terms of performance, it is not surprising that the GPU outperformed the CPU significantly, given a large enough batch size (Figure 6). We also observed the expected plateau in performance increase due to the saturation of the CUDA driver with simultaneously executing kernels. We explain the theoretical advantage of the parallelized rotamer sampler over naïve rotamer sampling in the Methods (Parallelization of Differentiable Rotamer Sampling with improved memory usage). Our energy function was not completely optimized since many of the pairwise computations were computed for both the upper and lower triangles of the distance matrix, resulting in a 2-fold redundancy in certain calculations that are symmetric in the particle order. We encountered difficulty with the limited memory on the GPU (the NVIDIA Tesla T4 only has 16 GB of video RAM), particularly with the energy function, whereas the CPU had access to 64 GB of RAM at the cost of compute speed. We did not perform benchmarks on the M1 Max GPU because the PyTorch backend for Apple’s Metal Performance Shaders is not complete.

### Analogy of BGs with traditional MD

In the case of classical molecular force fields, the high energy barriers tend to be a result of physical singularities in Lennard–Jones and Coulomb potentials at low distance due to internuclear forces, with Lennard–Jones repulsion ($\propto 1/{r}^{12})$ dominating any electrostatic force ($\propto 1/{r}^2$) at low distance. The enormous Lennard–Jones repulsion at low distances is due to the Coulomb barrier, from positively charged nuclei coming into contact; we must therefore avoid generating states in which nuclei significantly overlap. In traditional MD and in Monte Carlo methods, umbrella sampling is used to regularize the singularities; we implicitly implemented umbrella sampling in this work by regularizing the Lennard–Jones forces at low distances. In Noé *et al*. [14], a logarithmic regularization is performed on the total energy. In addition, the force field is further regularized by the neural network training process, such as through gradient clipping [36], dropout [37] and weight penalties [16]. Such methods of regularization are necessary for neural network convergence (also see Supplemental Methods, Avoiding Singularities in the Energy Function During Backpropagation); therefore, BGs as originally presented are umbrella sampled.

Despite their shortcomings, BGs maintain at least one significant advantage over traditional MD with umbrella sampling. BGs implicitly retain some memory of the entire training trajectory, and therefore they may reuse knowledge of the structure between different energy states. For example, an ideal BG may learn the correlations among the internal coordinates, correlations which may hold between distinct energy minima.

### Limitations and challenges

Significant challenges remain in the practical use of BGs. Even though we were able to reproduce RMSFs with high correlation (Figure 3C), the resulting structures still resemble the native state. We found that the energy landscape of the protein was a highly sensitive function of both the temperature and learning rate; fine-tuning of both appears to be required to produce useful results. However, the methods we present in this work make the fine-tuning process more easily accessible to researchers. Future work may also examine the effect of neural network architecture and other hyperparameters on angle generation since we did not study that effect here. Finally, it may be possible to further accelerate certain computations with a field-programmable gate array, which can be configured on a per-protein basis to perform rotamer sampling and energy computation.

In this work, we only performed limited benchmarking of a single protein, focusing on carefully analyzing the theoretical and practical improvements in BG methods. Extensive testing on other macromolecular systems and benchmarking with different force fields is necessary to draw solid conclusions about BGs as applied to MD. However, at the current stage, our primary focus was to establish the theoretical foundation for our approach and demonstrate its potential. The upcoming phases of our research will encompass the extensive testing required to solidify our method’s practical utility. In addition, our improvements did not address the issue of bias in choosing initial trainable parameters for a BG; i.e. we still needed to train the model to mimic a chosen input state before allowing it to explore the remainder of the configuration space (Results, Initializing Boltzmann Generators at the Native State). It is unknown at present if BG models can be made trainable and convergent without this initial bias.

Fortunately, it is simple to swap out the AMBER 14 force field we used in this model with other force field parameters, through the openmmforcefields package. In addition, our code is also written in a way so that arbitrary proteins (and other macromolecules) can be inserted into the energy function in the form of a PDB file and appropriate choice of force field, allowing for future benchmarking to be performed.

## CONCLUSION

In conclusion, we present a comprehensive toolkit of differentiable methods for molecular science. Our contributions include *ad hoc* propagation of forces from an arbitrary force field for cases in which rewriting the force field is infeasible, differentiable and parallel rotamer sampling/protein fragment assembly, a guide to writing molecular force fields in a differentiable programming framework, decoupling of energy and entropic estimation, and mathematical results on 3D point cloud alignment and 3D rotation representation that can be applied to problems in molecular geometry. We additionally address potential sources of bias in molecular structure generation and outline the approach to remaining sources of bias, which we did not implement. We demonstrate that our methods are efficiently implementable on CPU and GPU, and mathematically sound. We hope that other researchers will find these methods and the accompanying reference code useful in investigating molecular energy landscapes.

**Key Points**

- Boltzmann generator neural networks have emerged as an alternative to molecular dynamics.
- Existing Boltzmann generators are computationally inefficient and biased.
- We propose geometric methods that enable unbiased and direct sampling of the rotameric conformations of molecules such as proteins and RNAs.
- The methods described here may be useful in generating biologically relevant molecular conformations at high speed and accuracy.

## Supplementary Material

Movie_1_Chicken_villin_headpiece_bbad456

Movie_2-Bacteriocin_AS-48_bbad456

algorithms_bbad456

Supplemental_Methods_bbad456

Supplemental_Tables_bbad456

Trajectory_1_bbad456

Trajectory_2_bbad456

## DATA AND MATERIALS AVAILABILITY

All code (Jupyter notebooks, Python scripts) to reproduce the results in this work are included in a BitBucket repository (https://bitbucket.org/dokhlab/diffrot-manuscript/). Non-essential pretrained model weights are available on request to the corresponding author (dokh@psu.edu). We also include example movies generated by our rotameric Boltzmann generators (Movies S1 and S2).

## Author Biographies

**Congzhou M Sha** is an MD-PhD student at the Penn State College of Medicine, specializing in machine learning for molecular biophysics, structural biology, and drug docking.

**Jian Wang** is an Assistant Professor of Pharmacology at Penn State College of Medicine, applying machine learning and advanced algorithms for protein allostery, molecular dynamics, structural biology, drug docking, and other areas of computational biology.

**Nikolay V Dokholyan** is the G. Thomas Passananti Professor of Pharmacology at Penn State College of Medicine, with broad expertise in protein allostery and engineering, molecular dynamics, drug discovery, neurodegenerative disease, and machine learning.
