# Supplementary material for: Differentiable rotamer sampling with molecular force fields
Source: Brief Bioinform. 2023 Dec 12;25(1):bbad456. doi: 10.1093/bib/bbad456 (PMC10720392; doi:10.1093/bib/bbad456)
Supplement: algorithms_bbad456 [file algorithms_bbad456.pdf]

Algorithm 1 FFDiff: Differentiating arbitrary molecular force fields.

procedure FFDIFF(FF, f, θ)
  ▷ Inputs: Force field FF, an automatically differentiable function f which provides particle positions x, and trainable parameters θ
  Compute $x := f(\theta)$
  Compute forces on each particle $F := FF(x)$
  Compute $U_{new} := -F \cdot x$
  return AUTODIFF(U_{new}, θ) ▷ Use existing automatic differentiation code to compute gradients of U_{new} with respect to θ.
end procedure

Algorithm 2 PrepRot: Computing rotation parameters.

procedure PREPROT(B, P)
  ▷ Inputs: Molecular bonds (an undirected graph) B and positions P ($n \times 3$ array) of the macromolecular system.
  [Optional] $B :=$ DEPTHFIRSTSEARCH(B) ▷ Turn B into a tree.
  $L :=$ list(B) ▷ Organize all the molecular bonds into a list of edges, (a, b).
  for $(a, b) \in L$ **do**
    if deg(a) $= 1$ or deg(b) $= 1$ **then** ▷ Remove all bonds with trivial dihedral angle.
      Remove (a, b) from L
    end if
  end for
  $D :=$ dict() ▷ A dictionary/hashmap from bonds to a list of nodes
  for $(a, b) \in L$ **do**
    Remove (a, b) from B
    $C :=$ CONNECTEDCOMPONENTS(B) ▷ Find the connected components of the macromolecule system with the given bond cut.
    $c :=$ SMALLEST(C) ▷ Find the connected component with the fewest vertices.
    $O := P[a]$ ▷ Record the origin of rotation for the bond.
    axis $:=$ NORMALIZE($P[b] - P[a]$) ▷ Calculate the axis of rotation for the bond (a vector with unit length).
    $D[(a, b)] := (O, \text{axis}, c)$ ▷ Later, when we perform a rotation about the bond (a, b), we will rotate only the atoms in c.
    Add (a, b) to B ▷ Restore the bond in the graph.
  end for
  return D
end procedure

Algorithm 3 DiffRot: Differentiable dihedral rotation.

procedure DiffRot(Θ, P, D)
▷ Inputs: Dihedral angles Θ (an array of length d), starting positions of atoms P ($n \times 3$ array), and D data dictionary with d entries computed by Algorithm 2.
    for $(\theta, (O, \text{axis}, c)) \in \Theta \times D$ **do** ▷ Dihedral angle θ, rotation origin O, rotation axis axis, and list of atom indices c.
        $R :=$ AxisAngleToRotation(θ, axis) ▷ Use Rodrigues' formula or a similar method to determine the rotation matrix from an axis and angle.
        for $i \in c$ **do**
            $P[i] := P[i] - O$ ▷ Translate the center of rotation to the origin.
            $P[i] := RP[i]$ ▷ Perform the rotation about the axis (matrix multiplication).
            $P[i] := P[i] + O$ ▷ Translate the origin back to the center of rotation.
        end for
    end for
    return P
end procedure

Algorithm 4 Quaternion alignment

procedure QuaternionAlignment(X, Y)
▷ Inputs: two matrices of identified positions X and Y, each with shape $N \times 3$.
    $(\bar{X})_j = \frac{1}{N} \sum_i^N X_{ij}$ ▷ Find the centroid of each set of points.
    $(\bar{Y})_j = \frac{1}{N} \sum_i^N Y_{ij}$
    $(X_C)_{ij} := X_{ij} - \bar{X}_j$ ▷ Center each set of points.
    $(Y_C)_{ij} := Y_{ij} - \bar{Y}_j$
    $H[i, j] := \sum_k X_C[i, k] Y_C[j, k]$ ▷ Compute the covariance matrix
    Compute F ▷ The 4×4 symmetric matrix, as described in the Methods.
    $v_{max} :=$ LargestEigen(F) ▷ Compute the eigenvector corresponding to the largest magnitude eigenvalue, normalized by the squared norm on the components. We use repeated matrix squaring to accomplish this, in Algorithm 5.
    Compute R for the quaternion which $v_{max} := (q_0, q_1, q_2, q_3)$ represents. ▷ This equation may be found in Methods.
    return $R, \bar{X}, \bar{Y}$
end procedure

Algorithm 5 PowerIterWithSquaring: An implementation of LargestEigen through repeated matrix squaring.

procedure PowerIterWithSquaring(A, k)
  ▷ Input: an $N \times N$ matrix, and number of iterations.
  $A_1 := A$
  for $i \in [1, \cdots, k]$ **do**
    $A_{i+1} := A_i^2 / |A_i|$ ▷ Square and normalize A by an arbitrary matrix norm.
  end for
  $(v_{max})_i := A_{k+1}[i, 1]$ ▷ Choose an arbitrary column or row of A_{k+1}. This will be an eigenvector.
  $\hat{v}_{max} :=$ Normalize(v_{max}) ▷ Normalize the eigenvector by the norm induced by the Euclidean inner product, which coincides with the definition of a unit quaternion.
  return $\hat{v}_{max}$
end procedure

Algorithm 6 DiffRotParallel: Parallel differentiable dihedral rotation for proteins.

procedure DIFFROTPARALLEL(Θ, B, P, N_f)
▷ Inputs: Dihedral angles Θ (an array of length d), molecular bonds B, representing r residues, starting positions of atoms P ($n \times 3$ array), and number of fragments for parallelization N_f.
    B_{cut} := list()
    for $i \in [1, 2, \cdots, N_f]$ **do**
        b := select peptide bond between residues $(i \cdot \frac{r}{N_f} - 1)$ and $(i \cdot \frac{r}{N_f})$
        Add b to B_{cut}
        Remove b from B
    end for
    C := CONNECTEDCOMPONENTS(B)
    $Ps_0, Ds, \Theta s, P_N, PC$:= dict() ▷ Initialize all to empty dictionaries.
    for $b \in C$ **do** ▷ For each connected component in the resulting tree
        $Ps_0[b] := P[b]$ ▷ Get the subset of positions for the connected component
        $P_N[b] := b$[N-terminal]+ b_{prev}[C-terminal] ▷ Select the N-terminal N2 nitrogen and Cα of the first residue of the current fragment, as well as the C-terminal C1 carbon and carbonyl oxygen positions of the last residue of the previous fragment b_{prev}.
        $P_C[b] := b$[C-terminal]+ b_{next}[N-terminal] ▷ Similarly for the alignment of the next fragment.
        $Ds[b]$:= PREPROT($b, Ps_0[b]+ P_N[b] + P_C[b]$) ▷ Get the metadata for each connected component, using Algorithm 2. Append extra atoms from the N- and C-terminals for later alignment.
        $\Theta s[b] := \Theta[b]$ ▷ Select the appropriate number of dihedral angles from Θ to match the number of dihedrals in $Ds[b]$. Since we are training Θ, it does not matter how we partition, as long as it is consistent during training.
    end for
    Ps_{int} := dict()
    for $b, \theta_b, p_b, d_b \in C \times \Theta s \times P_b \times Ds$ **do**
        $Ps_{int}[b]$:= DIFFROT(θ_b, p_b, d_b) ▷ Apply dihedral rotations to each fragment individually.
    end for
    Ps_{final} := dict($1 => Ps_{int}[1]$)
    for $b, p_b \in C[2 \cdots N_f] \times Ps_{int}[2 \cdots N_f]$ **do** ▷ Build the chain from the fragments. b indexes the fragments in order from N- to C-terminal.
        $p_C := p_b[P_C[b-1]]$ ▷ Get the transformed positions of the C-terminal from the previous fragment and the N-terminal from the current fragment, which we will align.
        $p_N := p_b[P_N[b]]$
        $R, \bar{p}_C, \bar{p}_N$:= QUATERNIONALIGNMENT(p_C, p_N) ▷ Align the fragments, using the quaternion approach or the Kabsch algorithm.
        $Ps_{\text{final}}[b] := R(p_b - \bar{p}_C) + p_N$ ▷ Apply the transformation for optimal alignment.
        [Optional] Apply an additional dihedral rotation here for the bonds at which we cut. This will recover the remaining degrees of freedom.
    end for
    Ps := stack and trim Ps_{final} so that positions are indexed in the correct order
    return Ps ▷ Output: the $N \times 3$ matrix of positions for all atoms.
end procedure
