# Supplementary material for: Differentiable rotamer sampling with molecular force fields
Source: Brief Bioinform. 2023 Dec 12;25(1):bbad456. doi: 10.1093/bib/bbad456 (PMC10720392; doi:10.1093/bib/bbad456)
Supplement: Supplemental_Methods_bbad456 [file supplemental_methods_bbad456.docx]

# Supplemental Methods

## Ad hoc propagation of forces

In cases in which the force field is not automatically differentiable, we outline how to backpropagate gradients through the force field to the network.

Since MD force fields are optimized and parallelized to calculate *forces* in addition to energies, we propose using the forces directly as gradients for neural network training. Assume that a neural network $N$ produces atomic positions $\boldsymbol{x}$ from Gaussian random variables $\boldsymbol{z}$ and neural network parameters $\boldsymbol{p}$:

$$\boldsymbol{x}=N(\boldsymbol{z};\boldsymbol{p})$$

( 1 )

We sought to optimize $\boldsymbol{p}$ with respect to the energy function

$$U_{MD}\left( \boldsymbol{x} \right)=U(N\left( \boldsymbol{z};\boldsymbol{p} \right))$$

( 2 )

In other words, we sought to calculate the gradients

$$\frac{\partial U}{\partial p_{i}}=\sum_{j} \frac{\partial U}{\partial x_{j}}\cdot\frac{\partial x_{j}}{\partial p_{i}}=\sum_{j} \frac{\partial U}{\partial x_{j}}\cdot\frac{\partial\left( N\left( \boldsymbol{z};\boldsymbol{p} \right) \right)_{j}}{\partial p_{i}}$$

( 3 )

However, by definition, the forces are the gradients of the energy with respect to the position

$$F_{j}=-\frac{\partial U_{MD}}{\partial x_{j}}$$

( 4 )

Therefore, we do not need to backpropagate gradients through the external molecular force field to provide gradients with respect to $\boldsymbol{p}$. Instead of differentiating $U_{MD}$ to train the network, we propose to use the loss

$$U_{new}=-\sum_{j} F_{j}\cdot x_{j}=-\sum_{j} F_{j}\cdot\left( N\left( \boldsymbol{z};\boldsymbol{p} \right) \right)_{j}$$

( 5 )

Notice that $F_{j}$ is no longer a function of $\boldsymbol{x}$ (and therefore not of $\boldsymbol{z}$ or $\boldsymbol{p}$ either), since we are using it as a constant input from the molecular force field. Taking a derivative,

$$\frac{{\partial U}_{new}}{\partial x_{i}}=-\sum_{j} F_{j}\cdot\frac{\partial x_{j}}{\partial x_{i}}=-F_{i}=\frac{\partial U_{MD}}{\partial x_{i}}$$

( 6 )

Since the gradients for $U_{new}$ are the same as for $U_{MD}$, there is no theoretical difference between training on $U_{new}$ instead of on $U_{MD}$. There is no need for a statistical estimate (i.e. KL divergence) of the gradients with respect to the force field energy, as was done in the original work by Noé et al.^1^.

This method is shown in Algorithm 1: FFDiff.

## Physical interpretation of $U_{new}$

We can show that $U_{new}$ has a physical meaning beyond being a convenient loss function.

First, we note that $U_{new}$ is manifestly rotation-invariant. It is the sum of the inner products of vectors in Euclidean space; rotation of the coordinate system rotates both the position and force contravariantly, leaving the inner product unchanged.

Second, $U_{new}$ is translation invariant. Say that we translate a system of particles by a vector $\boldsymbol{p}$. Then in the new primed coordinates, $U_{new}^{'}$ is

$$U_{new}^{'}=-\sum_{j} F_{j}^{'}\cdot x_{j}^{'}=-\sum_{j} F_{j}\cdot{(x}_{j}-\boldsymbol{p})$$

( 7 )

assuming the intramolecular forces are unchanged ($F^{'}=F$) with translation ($x^{'}=x-\boldsymbol{p}$).

Splitting up the net forces $F_{j}$ into all pairwise forces $F_{ji}$ (the force exerted on particle $j$ by particle $i$), and then splitting up the sum, we may write

$$U_{new}^{'}=-\sum_{i>j} F_{ji}\cdot\left( x_{j}-\boldsymbol{p} \right)-\sum_{j>i} F_{ji}\cdot\left( x_{j}-\boldsymbol{p} \right)-\sum_{i=j} F_{ji}\cdot\left( x_{j}-\boldsymbol{p} \right)$$

( 8 )

By Newton’s third law (assuming the molecular force field is conservative), every force must be paired with an equal and opposite force, so $F_{ji}=-F_{ij}$, and therefore the third sum is 0 ($i=j$). The remaining sums are over the upper and lower triangles of $F_{ji}$.

$$U_{new}^{'}=-\sum_{i>j} F_{ji}\cdot\left( x_{j}-\boldsymbol{p} \right)-\sum_{j>i} F_{ji}\cdot\left( x_{j}-\boldsymbol{p} \right)$$

( 9 )

By linearity,

$$U_{new}^{'}=-\sum_{i>j} \left[ F_{ji}\cdot x_{j}-F_{ji}\cdot\boldsymbol{p} \right]-\sum_{j>i} \left[ F_{ji}\cdot x_{j}-F_{ji}\cdot\boldsymbol{p} \right]=-\sum_{i>j} F_{ji}\cdot x_{j}-\sum_{j>i} F_{ji}\cdot x_{j}-\boldsymbol{p}\cdot\sum_{i>j} F_{ji}-\boldsymbol{p}\cdot\sum_{j>i} F_{ji}=-\sum_{i>j} F_{ji}\cdot x_{j}-\sum_{j>i} F_{ji}\cdot x_{j}-\boldsymbol{p}\cdot\left( \sum_{i>j} \left( F_{ji}+F_{ij} \right) \right)$$

( 10 )

Finally, we apply Newton’s third law again so that the third term cancels, and therefore $U_{new}^{'}$ has no dependence on $\boldsymbol{p}$ and therefore it is translation-invariant.

To interpret the physical meaning of $U_{new}$, it suffices to examine a simple system. Say there is a pair of particles connected by a spring, with particle 1 at the origin ($x_{1}=[0, 0, 0])$ and particle 2 at $x_{2}=[1, 0, 0]$. Assume that the spring is compressed, so that the force pushes the particles away from each other: $F_{1}=[-k,0, 0]$ and $F_{2}=[k, 0, 0]$. In this case, we can directly calculate

$$U_{new}={-F}_{1}\cdot x_{1}-F_{2}\cdot x_{2}=-\left[ -k, 0, 0 \right]\cdot\left[ 0, 0, 0 \right]-\left[ k, 0, 0 \right]\cdot\left[ 1, 0, 0 \right]=-k$$

( 11 )

Hence, if the spring is instead stretched beyond its equilibrium length, then $U_{new}$ is positive. Since $U_{new}$ is invariant under rotation and translation of the system, our choice of coordinate system was irrelevant. Generalizing to many pairs of particles and many springs, the interpretation for $U_{new}$ is that it measures the total compressive energy of the system, with negative values indicating the system is compressed with respect to equilibrium and positive values indicating the system is stretched with respect to equilibrium.

## Rewriting molecular force fields to be end-to-end differentiable

After it is prepared, the PyTorch energy function requires the user only to supply the coordinates (in nanometers) of each atom, in the same order as presented in the input molecular file. From the user-supplied atomic positions $x_{i}$, we calculate all pairwise displacements

$$s_{ij}=x_{i}-x_{j}$$

( 12 )

and Euclidean distances

$$d_{ij}=\left| s_{ij} \right|=\left| x_{i}-x_{j} \right|=\sqrt{\sum_{k} \left( \left( x_{i} \right)_{k}-\left( x_{j} \right)_{k} \right)^{2}}$$

( 13 )

For the all-atom AMBER 14 force field^2^, we implemented: (1) harmonic bond lengths, (2) harmonic bond angles, (3) pairwise atomic Coulomb interactions, (4) pairwise atomic Lennard-Jones potentials, (5) periodic backbone dihedral (torsional) angle energies, and (6) generalized Born implicit solvent energies. In our framework, force fields are plug-and-play, and alternative and more recent force fields are supported through OpenMM’s openmmforcefields package.

First, we imported the molecular topology into OpenMM, which assigns parameter values for each atom or tuple of atoms. Second, we referenced the OpenMM documentation to implement each of the forces.

Harmonic bond lengths: the AMBER force field provides particle indices representing a covalent bond $b=\left( i,j \right)$, the bond length $l_{b}$, and the bond strength $k_{b}$. For each bond, we calculate an energy term with these parameters and the pairwise atomic distances:

$$E_{harmonic bonds}=\sum_{b} \frac{1}{2}k_{b}\left( d_{ij}-l_{b} \right)^{2}$$

( 14 )

In OpenMM, the parameters are contained in a HarmonicAngleForce object.

Harmonic bond angles: for every triple of atoms which are covalently connected in a linear chain, the AMBER force field provides particles indices $t=\left( i,j,k \right)\in T$ in which $j$ is the middle atom, a bond angle $\phi_{t}$, and a bond angle strength $k_{t}$. We calculate the angle $\theta_{ijk}$ formed between $s_{ji}$ and $s_{jk}$ (the order of the indices is important) through the dot product identity

$$\cos\theta_{ijk}=\frac{s_{ji}\cdot s_{jk}}{\left| s_{ji} \right|\left| s_{jk} \right|}=\frac{s_{ji}\cdot s_{jk}}{d_{ji}d_{jk}}$$

( 15 )

The corresponding energy term is then:

$$E_{harmonic angles}=\sum_{t} \frac{1}{2}k_{t}\left( \theta_{ijk}-\phi_{t} \right)^{2}$$

( 16 )

In OpenMM, the parameters are contained in a HarmonicBondForce object.

Pairwise atomic Coulomb and Lennard-Jones interactions: for every pair of atoms, there is an electrostatic force as a function of the interatom distance $d_{ij}$ due to estimated partial charges $q_{i}$ from AMBER.

$$E_{electrostatic}=\sum_{i<j} \frac{q_{i}q_{j}}{4\pi\epsilon_{0}d_{ij}}$$

( 17 )

For every pair of atoms, there is a Lennard-Jones potential which approximates long-range dipole-dipole attractions and short-range nuclear repulsions. AMBER provides an energy scale $\epsilon_{ij}$ and interaction distance $\sigma_{ij}$ for each pair of particles.

$$E_{LJ}=\sum_{i<j} \frac{\epsilon_{ij}}{4}\left[ \left( \frac{\sigma_{ij}}{d_{ij}} \right)^{12}-\left( \frac{\sigma_{ij}}{d_{ij}} \right)^{6} \right]$$

( 18 )

Certain Lennard-Jones and Coulomb interactions are often ignored or modified for atoms which are within a certain number of bonds of each other. These modifications can be thought of as setting elements of the matrices $\epsilon_{ij}$, $\sigma_{ij}$, and $d_{ij}$ to specific values and are included in the OpenMM implementation of AMBER.

During regularization of the force field, we adjusted the Lennard-Jones potential so that for all particles below a certain distance, the force was a constant repulsive one. Given a maximum force magnitude for internuclear repulsion $F_{m}$, and for each Lennard-Jones interaction, we first computed the magnitude of the force for that interaction

$$F_{LJ,ij}(d_{ij})=\frac{\epsilon_{ij}}{4d_{ij}}\left[ 6\left( \frac{\sigma_{ij}}{d_{ij}} \right)^{6}-12\left( \frac{\sigma_{ij}}{d_{ij}} \right)^{12} \right]$$

( 19 )

For numerical stability, we computed $\left( \frac{\sigma_{ij}}{d_{ij}} \right)^{6}/d_{ij}$ and $\left( \frac{\sigma_{ij}}{d_{ij}} \right)^{12}/d_{ij}$ in the given order of operations rather than taking the ratio of the powers $\frac{\sigma_{ij}^{6}}{d_{ij}^{7}}$ and $\frac{\sigma_{ij}^{12}}{d_{ij}^{13}}$. At $r_{min,ij}=\sqrt[6]{2}\sigma$, the Lennard-Jones interaction achieves its minimum value, and thus $F_{LJ,ij}\left( r_{min,ij} \right)=0$. This is the only minimum of the Lennard-Jones potential, and it can be seen that $F_{LJ,ij}$ is a monotonically increasing function of the distance, with an asymptote at $d_{ij}=0$ which tends to negative infinity. Therefore, to find the value $r_{max grad, ij}$ at which the magnitude of the repulsive force is equal to $F_{m}$, we used the bisection method for root finding^3^. We did not use the Newton-Raphson method or other methods which require the second derivative of the Lennard-Jones potential due to numerical stability.

For a given value of ${0\leq F}_{m}<\infty$, we know that the solution of the equation

$$\left| F_{LJ,ij}\left( d_{ij} \right) \right|-F_{m}=0$$

( 20 )

lies in the interval $(0, r_{min,ij}]$. Therefore by starting with the initial guess $d_{ij}^{0}=r_{min,ij}/2$, we may bisect the interval repeatedly to determine the approximate value of the zero, depending on if $\left| F_{LJ,ij}\left( d_{ij} \right) \right|>F_{m}$ or $\left| F_{LJ,ij}\left( d_{ij} \right) \right|<F_{m}.$ We performed this bisection $n_{bisect}=100$ times, which should ensure a negligibly small error for $r_{max grad, ij}$. Since $\sigma_{ij}$ was measured on the nanometer scale and numerical values for $\sigma_{ij}$, the bisection method should be accurate to roughly $1$ part in $2^{n_{bisect}}$, or ${10}^{-30}$ nanometers.

We defined the regularized energy as

$$E_{LJ,reg}=\sum_{i<j} \left\{ \begin{aligned} \frac{\epsilon_{ij}}{4}\left[ \left( \frac{\sigma_{ij}}{d_{ij}} \right)^{12}-\left( \frac{\sigma_{ij}}{d_{ij}} \right)^{6} \right], &\text{if} d_{ij}>r_{max grad, ij} \\ E_{0,ij}-F_{m}d_{ij}, &\text{otherwise} \end{aligned} \right.$$

( 21 )

Where $E_{0,ij}$ was chosen so that the $E_{LJ,reg}$ is continuous at $d_{ij}=r_{max grad,ij}$. By inspection, the derivative of the regularized potential at close internuclear distances is $-F_{m}$, as desired.

In OpenMM, parameters for Coulomb and Lennard-Jones interactions, as well as any modifications, are contained in a NonbondedForce object.

Periodic backbone dihedral (torsional) angle energies: for every linear chain of four atoms $c=(i,j,k,l)$, AMBER provides a periodic force for the dihedral angle, which is defined as the angle that $s_{ji}$ makes with respect to $s_{kl}$ when viewed in the plane whose normal is $s_{jk}$, defined by a periodicity $n_{c}$, energy scale $\epsilon_{c}$, and phase shift $\phi_{c}$.

The dihedral angle $\theta_{c}$ is calculated by first normalizing $s_{jk}$:

$$\hat{s}_{jk}=\frac{s_{jk}}{d_{jk}}$$

( 22 )

and projecting $s_{ji}$ and $s_{kl}$ onto the plane of interest:

$$\tilde{s}_{ji}=\left( 1-s_{ji}\cdot\hat{s}_{jk} \right)s_{ji}$$

$$\tilde{s}_{kl}=\left( 1-s_{kl}\cdot\hat{s}_{jk} \right)s_{kl}$$

( 23 )

Then the dot product identity states that

$$\tilde{s}_{ji}\cdot\tilde{s}_{kl}=|\tilde{s}_{ji}||\tilde{s}_{kl}|\cos\theta_{c}$$

( 24 )

To avoid numerical instabilities and to recover the dihedral angle from the full range $[-\pi, \pi)$,^4^ we compute

$$\left( \hat{s}_{jk}\times\tilde{s}_{ji} \right)\cdot\tilde{s}_{kl}=|\tilde{s}_{ji}|\left| \tilde{s}_{kl} \right|\sin\theta_{c}$$

( 25 )

which is a special case of the polar sine, and finally

$$\theta_{c}=\text{atan2}\frac{\tilde{s}_{ji}\cdot\tilde{s}_{kl}}{\left( \hat{s}_{jk}\times\tilde{s}_{ji} \right)\cdot\tilde{s}_{kl}}$$

( 26 )

The torsional energy is then:

$$E_{torsion}=\sum_{c} \epsilon_{c}\left[ 1+\cos\left( n_{c}\theta_{c}-\phi_{c} \right) \right]$$

( 27 )

In OpenMM, the parameters are contained in a PeriodicTorsionForce object.

Generalized Born implicit solvent: we used the generalized Born implicit solvent with improved parameters^5–7^, labeled GBn2 in OpenMM or igb=8 in AMBER. The functional form for this energy is complicated, so we only present the calculations necessary to reproduce the energy. Global parameters for the implicit solvent are $\text{cutoff}_{neck}=0.68$, $\text{scale}_{neck}=0.826836$, $\text{offset}=0.0195141$, $\epsilon_{solute}=1$, $\epsilon_{solvent}=78.5$, and integral corrections $\eta=28.3919551$ and $\theta=0.14$; per particle and per pair parameters are Born radii $\text{radius}_{i}$, Born/van der Waals cutoff adjustments $\text{or}_{i}$ and $\text{sr}_{i}$, partial charges $q_{i}$, effective radii scaling parameters ($\alpha_{i},\beta_{i},\gamma_{i})$, and pairwise neck integral parameters $d_{0,ij}$ and $m_{0, ij}$. The letters $i$ and $j$ are particle indices. Given pairwise particle distances $d_{ij},$ we calculate

$$D_{ij}=d_{ij}-\text{sr}_{j}$$

( 28 )

$$L_{ij}=max(\text{or}_{i}, D_{ij})$$

( 29 )

$$U_{ij}=d_{ij}+\text{sr}_{j}$$

( 30 )

$$I_{vdw, ij}=\left\{ \begin{aligned} \frac{1}{2}\left[ \frac{1}{L_{ij}}-\frac{1}{U_{ij}}+\frac{1}{4}\left( d_{ij}-\frac{\text{sr}_{j}^{2}}{d_{ij}} \right)\left( \frac{1}{U_{ij}^{2}}-\frac{1}{L_{ij}^{2}} \right)+\frac{1}{2d_{ij}}\text{log}\frac{L_{ij}}{U_{ij}} \right], &d_{ij}>\text{sr}_{i}-\text{or}_{i} \\ 0, &\text{otherwise} \end{aligned} \right.$$

( 31 )

$$I_{neck, ij}=\left\{ \begin{aligned} \frac{m_{0, ij}}{1+100\left( d_{ij}-d_{0,ij} \right)^{2}+300,000\left( d_{ij}-d_{0,ij} \right)^{6}}, &d_{ij}<\text{radius}_{i}+\text{radius}_{j}+\text{cutoff}_{neck} \\ 0, &\text{otherwise} \end{aligned} \right.$$

( 32 )

$$I_{ij}=I_{vdw,ij}+\text{scale}_{neck}I_{neck, ij}$$

( 33 )

For each particle, note that $I_{ii}=0$. We then compute

$$I_{i}=\sum_{j} I_{ij}$$

( 34 )

$$\psi_{i}=I_{i}\text{or}_{i}$$

( 35 )

$$B_{i}=\left[ \frac{1}{\text{or}_{i}}-\frac{\tanh\left( \alpha_{i}\psi_{i}-\beta_{i}\psi_{i}^{2}+\gamma_{i}\psi_{i}^{3} \right)}{\text{radius}_{i}} \right]^{-1}$$

( 36 )

$$\text{or}_{\text{offset},i}=\text{or}_{i}+\text{offset}$$

( 37 )

$$f_{GB,ij}=\sqrt{d_{ij}^{2}+B_{i}B_{j}\exp\left( -\frac{d_{ij}^{2}}{4B_{i}B_{j}} \right)}$$

( 38 )

The implicit solvent energy is then

$$E_{GB}=-\frac{1}{2}\sum_{i} \frac{q_{i}^{2}}{4\pi\epsilon_{0}B_{i}}\left[ \frac{1}{\epsilon_{solute}}-\frac{1}{\epsilon_{solvent}} \right]+\sum_{i} \eta\left( \text{or}_{\text{offset},i}+\theta\right)^{2}\left( \frac{\text{or}_{\text{offset},i}}{B_{i}} \right)^{6}-\sum_{i<j} \frac{q_{i}q_{j}}{4\pi\epsilon_{0}f_{GB,ij}}\left[ \frac{1}{\epsilon_{solute}}-\frac{1}{\epsilon_{solvent}} \right]$$

( 39 )

In OpenMM, the parameters are contained in a CustomGBForce object. The implicit solvent modifies the Coulomb energies computed earlier.

We computed all non-indexed sums on pairs of particles using the full matrix format, which duplicated some of the necessary computations.

*Avoiding singularities in the energy function during backpropagation*

Numerical singularities in gradients arose due to true singularities in the energy function and artificial singularities due to the computational graph.

True singularities in the gradient arose during both the forward and backward passes due to square roots, logarithms, and negative powers. In all cases in our energy function, we are guaranteed that the argument of the function is nonnegative. Therefore, we avoided true singularities by adding a small positive offset $\epsilon$ to all affected computations.

For computations which were performed but later discarded, such as those involving the diagonal of the distance matrix, we manually set specific terms to fixed constant values using PyTorch’s masked fill function so that during backpropagation, the gradient for those terms would be fixed to 0 instead of NaN.

## The Kabsch algorithm and a quaternionic alternative

Given two sets of positions $x$ and $y$, represented by $N\times3$ matrices $x_{ij}$ and $y_{ij}$, with $i$ labeling the particle number and $j$ labeling the 3D components, the goal is to find the best fit rotation matrix and translation which transform the positions of the particles in $x$ to the corresponding positions in $y$.

We first center the two sets of points, by averaging the 3D components over all particles

$$\bar{x}_{j}=\frac{1}{N}\sum_{i} x_{ij}$$

$$\bar{y}_{j}=\frac{1}{N}\sum_{i} y_{ij}$$

$$X_{ij}=x_{ij}-\bar{x}_{j}$$

$$Y_{ij}=y_{ij}-\bar{y}_{j}$$

( 40 )

We then calculate the covariance matrix

$$H_{ij}=\sum_{k} X_{ki}Y_{kj}$$

( 41 )

In the Kabsch method^8^, we calculate the singular value decomposition

$$H=U\Sigma V^{T}$$

( 42 )

Since the Kabsch method may result in improper rotations, we correct for changes in basis orientation by calculating

$$d=\text{sign}\left( \det\left( VU^{T} \right) \right)$$

( 43 )

and the optimal rotation matrix is expressed as

$$R=V\left[ \begin{matrix} 1 & 0 & 0 \\ 0 & 1 & 0 \\ 0 & 0 & d \end{matrix} \right]U^{T}$$

( 44 )

In the quaternionic approach^9,10^, the quaternion associated with the optimal rotation in 3D is the eigenvector $[q_{0},q_{1},q_{2},q_{3}]$ associated with the largest magnitude eigenvalue of the traceless, symmetric matrix

$$F=\left[ \begin{matrix} H_{11}+H_{22}+H_{33} & H_{23}-H_{32} & H_{31}-H_{13} & H_{12}-H_{21} \\ H_{23}-H_{32} & H_{11}-H_{22}-H_{33} & H_{12}+H_{21} & H_{13}+H_{31} \\ H_{31}-H_{13} & H_{12}+H_{21} & -H_{11}+H_{22}-H_{33} & H_{23}+H_{32} \\ H_{12}-H_{21} & H_{13}+H_{31} & H_{23}+H_{32} & -H_{11}-H_{22}-H_{33} \end{matrix} \right]$$

( 45 )

To convert the quaternion $q=(q_{0},q_{1},q_{2},q_{3})$ to a rotation matrix, we compute

$$R=\left[ \begin{matrix} q_{0}^{2}+q_{1}^{2}-q_{2}^{2}-q_{3}^{2} & 2\left( q_{1}q_{2}-q_{0}q_{3} \right) & 2\left( q_{1}q_{2}+q_{0}q_{2} \right) \\ 2(q_{1}q_{2}+q_{0}q_{3}) & q_{0}^{2}-q_{1}^{2}+q_{2}^{2}-q_{3}^{2} & 2(q_{2}q_{3}-q_{0}q_{1}) \\ 2\left( q_{1}q_{3}-q_{0}q_{2} \right) & 2\left( q_{2}q_{3}+q_{0}q_{1} \right) & q_{0}^{2}-q_{1}^{2}-q_{2}^{2}+q_{3}^{2} \end{matrix} \right]$$

( 46 )

In the quaternionic approach, the sign of the eigenvalue determines the orientation of the rotation. We assumed that the ideal proper rotation for our uses always has the largest magnitude eigenvalue.

## Technical details of parallelized differentiable rotamer sampling

We split the protein chain into $N_{f}$ fragments, splitting at the peptide bond between the C1 carbon of residue $i$ and the N2 nitrogen of residue $i+1$. We recorded the positions of atoms in each segment, $x_{k}^{f}$, where $f$ labels the fragment and $k$ labels the number of the atom. For the N-terminal of fragment $f$, we appended the positions of four atoms near the peptide bond (C1=O from the C-terminal residue of fragment $f-1$ and N2-C$\alpha$ from the N-terminal residue of fragment $f$) and appended those four positions to the $x_{k}^{f}$; we selected the same four atoms from the C-terminal of fragment $f$(C1=O from the C-terminal residue of fragment $f$ and N2-C$\alpha$ from the N-terminal residue of fragment $f+1$). Next, we applied our original rotamer sampling method on each fragment independently. We then aligned the C-terminal of $f$ to the N-terminal of $f+1$, thus joining the fragments together. Finally, we applied dihedral rotations using Rodrigues’ formula^11^ as discussed previously to the peptide bonds at which we initially split the protein chain, thus recovering the $N_{f}-1$ degrees of freedom lost upon splitting the chain into $N_{f}$ pieces.

## Memory advantage of parallelizing the differentiable rotamer sampling method

In addition to allowing for parallel computation of rotamer sampling, our improved approach significantly reduces memory usage. In our original dihedral sampling method, instead of transforming each position separately for each dihedral angle, we transformed all positions in the protein simultaneously, and then masked those positions which were not part of the associated connected component $CC(e_{m})$.

Assuming that PyTorch’s JIT compiler was not able to optimize the resulting computational graph, this approach could be memory-intensive and computationally inefficient, with roughly $N_{dih}\times N_{atoms}$ 3D matrix-vector multiplications and $2\times N_{dih}\times N_{atoms}$ 3D vector additions. In backpropagation, each of the intermediate matrices must be used, resulting in $N_{dih}\times N_{atoms}\times3$ floating point numbers being stored (a copy of the position matrix for each dihedral angle). In the parallel approach, ignoring the extra positions appended to each fragment, the number of dihedrals per fragment is roughly $\frac{N_{dih}}{N_{f}}$, and the number of positions per fragment is similarly $\frac{N_{atoms}}{N_{f}}$. The total number of floating point operations is therefore $N_{f}\times\left( \frac{N_{dih}}{N_{f}}\times\frac{N_{atoms}}{N_{f}} \right)=\frac{N_{dih}N_{atoms}}{N_{f}}$, so we have reduced the raw number of operations by a factor of $N_{f}$. The analogous calculation for memory usage shows a reduction by a factor of $N_{f}$. Assuming PyTorch launches and runs all $N_{f}$ kernels simultaneously on a GPU, we can achieve a speed-up over the original dihedral sampler approach of $N_{f}^{2}$ for large proteins, while using $\frac{1}{N_{f}}$ times as much memory.

## A simplified demonstration of biased sampling due to a discontinuous mapping

To show mathematically that the neural network training is biased in the latter case, even for values of $\theta$ within the range $[\theta_{max},\theta_{min}]$, we model the training process as a Markov chain^12^, and we also assume that $\theta$ can only take on a discrete set of values in the range $[\theta_{max},\theta_{min}]$ of size $N$, or the values $\theta_{min}-\epsilon$ or $\theta_{max}+\epsilon$. As a toy model, we assume that the training algorithm makes a transition $\theta\to\theta-\epsilon$ or $\theta\to\theta+\epsilon$ with equal probability while $\theta$ is within the range, and by the probability $1$ transitions

$$\theta_{min}-\epsilon\to\theta_{min}+n\epsilon$$

$$\theta_{max}+\epsilon\to\theta_{max}-n\epsilon$$

( 47 )

where $n$ represents the learning rate and $n<N/2$. This Markov chain is aperiodic and irreducible.

At equilibrium, the Markov chain converges to a stationary or invariant probability distribution on the angles, $\pi(\theta_{i})$, which satisfies the global balance equations. The invariant distribution can be calculated as the largest left eigenvalue/eigenvector of the probability transition matrix $q$, in which the $(i,j)$ entry is the probability of transitioning from state $i$ to state $j$.

For concreteness, we illustrate within an example. For the states

$$\theta_{i}=\{\theta_{min}-\epsilon,\theta_{min}, \theta_{min}+\epsilon,\theta_{min}+2\epsilon= \theta_{max}-2\epsilon,\theta_{max}-\epsilon,\theta_{max}, \theta_{max}+\epsilon\}$$

( 48 )

and transition matrix

$$q=\left[ \begin{matrix} 0 & 0 & 1 & 0 & 0 & 0 & 0 \\ \frac{1}{2} & 0 & \frac{1}{2} & 0 & 0 & 0 & 0 \\ 0 & \frac{1}{2} & 0 & \frac{1}{2} & 0 & 0 & 0 \\ 0 & 0 & \frac{1}{2} & 0 & \frac{1}{2} & 0 & 0 \\ 0 & 0 & 0 & \frac{1}{2} & 0 & \frac{1}{2} & 0 \\ 0 & 0 & 0 & 0 & \frac{1}{2} & 0 & \frac{1}{2} \\ 0 & 0 & 0 & 0 & 1 & 0 & 0 \end{matrix} \right]$$

( 49 )

the invariant distribution is

$$\pi=\left[ \begin{matrix} \frac{1}{18} & \frac{1}{9} & \frac{2}{9} & \frac{2}{9} & \frac{2}{9} & \frac{1}{9} & \frac{1}{18} \end{matrix} \right]$$

( 50 )

We see explicitly that values of $\theta$ outside the range $[\theta_{max},\theta_{min}]$ are discouraged $(\pi_{1}=\pi_{7}=\frac{1}{18}$) and that $\theta_{min}$ and $\theta_{max}$ are less likely than other values of $\theta$ which lie strictly within the range ($\frac{1}{9}$ vs $\frac{2}{9}$). In practice, there may be other transitions from $\theta$ outside the range to $\theta$ within the range, which is controlled by the learning rate. For example, replacing $q_{12}=q_{13}=q_{76}=q_{75}=\frac{1}{2}$ and $q_{1i}=q_{7i}=0$ for all other $i$ in Eq. ( 49 ) leads to

$$\pi\approx\left[ \begin{matrix} 0.053 & 0.105 & 0.210 & 0.263 & 0.210 & 0.105 & 0.053 \end{matrix} \right]$$

( 51 )

in which there is even more asymmetry among the states within the range.

## Estimation of temperature of Boltzmann-generated samples

We assumed that the energies of Boltzmann-generated samples follow an exponential distribution above the energy minimum^13^

$$E(x)\sim e^{-\beta x}$$

( 52 )

To reduce the influence of outliers on our estimate, we used the median energy of the sampled states as an estimator of the inverse temperature $\beta$. It is known analytically^14^ that

$${\Delta E}_{median}=E_{median}-E_{min}=\frac{\log2}{\beta}$$

( 53 )

We estimated $E_{min}$ by using the minimum energy of all generated structures.

## Additional useful identities

To aid in debugging of rotation matrices, we made use of the formula for the rotation angle and axis of an arbitrary $3\times3$ rotation matrix $R$.

$$\theta=\cos^{-1} \left( \frac{\text{tr} R-1}{2} \right)$$

( 54 )

$$\text{axis}=\frac{1}{2\sin\theta}\left[ \begin{matrix} R_{32}-R_{23} & R_{13}-R_{31} & R_{21}-R_{12} \end{matrix} \right]^{T}$$

( 55 )

where $\text{tr}$ is the trace. These identities are direct consequences of Rodrigues’ rotation formula.

## Learning rate tuning

To ensure convergent training, we found that a learning rate of $\gamma={10}^{-5}$ was necessary to prevent divergence during training due to large gradients. To arrive at this value of $\gamma$, we backpropagated gradients from the force field and entropic terms separately to the angles output by the neural network (i.e., only backpropagation only through the rotameric sampling and entropy estimation portions of the computational graph). We monitored these gradients over the course of a training session and found that angular gradients had a magnitude on the order of ${10}^{3}$. Therefore, parameters are tuned at a rate of approximately ${10}^{3}\cdot\gamma$, per epoch, ignoring the momentum contributions in Adam^15^. With the choice $\gamma={10}^{-5}$, we expect parameter tuning at a rate of roughly 0.01 per epoch; along with the choice of gradient clipping at 10.0, we enforced a large dynamic range of gradients with magnitudes under 0.01 to 10.0.

## Neural network architecture

We used simple feedforward neural networks as our Boltzmann generators, with 32 latent variables, 10 layers of 128 hidden units, dropout with a rate of 0.3^16^, residual connections^17^ between every other hidden layer, and LeakyReLU^18,19^ activations with a coefficient of 0.3. The final output layer dimension equal to the total number of dihedrals we wished to sample.

## Traditional molecular dynamics

We performed traditional MD using the AMBER 14 force field^2^ with generalized Born implicit solvent^5–7,20,21^ in OpenMM^22^. We used a Langevin thermostat at 310 K, friction coefficient of $91 \text{ps}^{-1}$, and timestep of $1 \text{fs}$. We simulated ${10}^{6}$ timesteps (1 ns trajectory), and recorded positions of all atoms every 1,000 timesteps.

## Order parameters

We calculated the root mean square deviation (averaged over the entire structure) and root mean square fluctuation (averaged over a trajectory per alpha-carbon), with respect to reference structure positions $x_{i,0}$.

$$\text{RMSD}(\{x_{i}\})=\sqrt{\frac{1}{N_{atoms}}\sum_{i} \left| x_{i}-x_{i,0} \right|^{2}}$$

$$\text{RMSF}\left( \left\{ x_{i} \right\} \right)_{j}=\sqrt{\frac{1}{N_{timesteps/samples}}\sum_{t} \left| x_{j}\left( t \right)-x_{j,0} \right|^{2}}$$

## Implementation details

All algorithms were implemented in Python 3.10 with PyTorch 1.13^23^. We loaded molecular topology and geometry using OpenMM 7.7^22^. We used PDBFixer 1.8.2^22^ to fix errors in PDB files and model hydrogens. We used NetworkX 2.8.4^24^ for all graph algorithms. We used a batch size of 8, and the Adam optimizer^15^, with learning rate $\gamma={10}^{-5}$, momentum coefficients $\beta_{1}=0.9$, $\beta_{2}=0.999$, and machine tolerance offset $\epsilon={10}^{-8}$. We also used a loss weight of $\gamma_{weight}={10}^{-2}$ for weight decay regularization.

For the molecular force field, we used AMBER 14 parameters^2^ as provided by OpenMM^22^, and the implicit generalized Born solvent (GBn2)^5–7,25^. Other packages used include NumPy 1.23.4^26,27^ and Pandas 1.5.2^28^ for data organization, Matplotlib 3.6.2^29^ for plotting, PyMol 2.5.0^30^ and Mol*^31^ for macromolecule visualization, and SciPy 1.9.3^32^. Training of models was performed in float32 accuracy on the CPU only of an M1 Max MacBook Pro with 64 GB RAM. Benchmarking was also performed on a single NVIDIA Tesla T4 GPU with 16 GB RAM on a Linux system. For reproducibility, we also set the random seed for PyTorch to 0.

To optimize the speed of dihedral angle application, we used static data structures and used PyTorch’s just-in-time compilation on the dihedral sampling function, as well as the generative neural networks. This technique allows PyTorch to optimize numerical array operations through fusion of kernels and memory locality optimizations.

# References

1. Noé, F., Olsson, S., Köhler, J. & Wu, H. Boltzmann generators: Sampling equilibrium states of many-body systems with deep learning. *Science (1979)* **365**, (2019).

2. Wang, J., Wolf, R. M., Caldwell, J. W., Kollman, P. A. & Case, D. A. Development and testing of a general Amber force field. *J Comput Chem* **25**, 1157–1174 (2004).

3. Burden, A., L. Burden, R. & Douglas Faires, J. *Numerical Analysis, 10th ed.* *Communications of the ACM* vol. 10 (2016).

4. Praxeolytic. Dihedral/torsion angle from four points in Cartesian coordinates in Python. *Stack Overflow* Preprint at https://stackoverflow.com/a/34245697 (2015).

5. Onufriev, A. V. & Case, D. A. Generalized Born Implicit Solvent Models for Biomolecules. *Annual Review of Biophysics* vol. 48 Preprint at https://doi.org/10.1146/annurev-biophys-052118-115325 (2019).

6. Mongan, J., Case, D. A. & McCammon, J. A. Constant pH molecular dynamics in generalized Born implicit solvent. *J Comput Chem* **25**, (2004).

7. Nguyen, H., Roe, D. R. & Simmerling, C. Improved generalized born solvent model parameters for protein simulations. *J Chem Theory Comput* **9**, (2013).

8. Kabsch, W. A solution for the best rotation to relate two sets of vectors. *Acta Crystallographica Section A* **32**, (1976).

9. Horn, B. K. P. Closed-form solution of absolute orientation using unit quaternions. *Journal of the Optical Society of America A* **4**, (1987).

10. Coutsias, E. A., Seok, C. & Dill, K. A. Using quaternions to calculate RMSD. *J Comput Chem* **25**, (2004).

11. Dai, J. S. Euler–Rodrigues formula variations, quaternion conjugation and intrinsic connections. *Mech Mach Theory* **92**, 144–152 (2015).

12. Gagniuc, P. *Markov Chains: From Theory to Implementation and Experimentation*. (Wiley, 2017).

13. Reif, F. & Scott, H. L. *Fundamentals of Statistical and Thermal Physics*. *American Journal of Physics* vol. 66 (McGraw Hill, 1998).

14. Ross, S. *Introduction to Probability and Statistics for Engineers and Scientists*. (Elsevier, 2021). doi:10.1016/C2018-0-02166-0.

15. Kingma, D. P. & Ba, J. L. Adam: A method for stochastic optimization. in *3rd International Conference on Learning Representations, ICLR 2015 - Conference Track Proceedings* (2015).

16. Srivastava, N., Hinton, G., Krizhevsky, A., Sutskever, I. & Salakhutdinov, R. Dropout: A simple way to prevent neural networks from overfitting. *Journal of Machine Learning Research* **15**, 1929–1958 (2014).

17. He, K., Zhang, X., Ren, S. & Sun, J. Deep residual learning for image recognition. in *Proceedings of the IEEE Computer Society Conference on Computer Vision and Pattern Recognition* vols 2016-Decem 770–778 (2016).

18. Xu, B., Wang, N., Chen, T. & Li, M. Empirical Evaluation of Rectified Activations in Convolutional Network. *ArXiv* (2015).

19. Maas, A. L., Hannun, A. Y. & Ng, A. Y. Rectifier nonlinearities improve neural network acoustic models. in *in ICML Workshop on Deep Learning for Audio, Speech and Language Processing* (2013).

20. Onufriev, A., Bashford, D. & Case, D. A. Exploring Protein Native States and Large-Scale Conformational Changes with a Modified Generalized Born Model. *Proteins: Structure, Function and Genetics* **55**, (2004).

21. Tsui, V. & Case, D. A. Theory and applications of the Generalized Born solvation model in macromolecular simulations. *Biopolymers* **56**, (2000).

22. Eastman, P. *et al.* OpenMM 7: Rapid development of high performance algorithms for molecular dynamics. *PLoS Comput Biol* **13**, (2017).

23. Paszke, A. *et al.* PyTorch: An imperative style, high-performance deep learning library. in *Advances in Neural Information Processing Systems* vol. 32 (2019).

24. Hagberg, A. A., Schult, D. A. & Swart, P. J. Exploring network structure, dynamics, and function using NetworkX. in *7th Python in Science Conference (SciPy 2008)* (2008).

25. Bondi, A. van der Waals Volumes and Radii. *J Phys Chem* **68**, 441–451 (1964).

26. Harris, C. R. *et al.* Array programming with NumPy. *Nature* vol. 585 357–362 Preprint at https://doi.org/10.1038/s41586-020-2649-2 (2020).

27. Van Der Walt, S., Colbert, S. C. & Varoquaux, G. The NumPy array: A structure for efficient numerical computation. *Comput Sci Eng* **13**, 22–30 (2011).

28. McKinney, W. Data Structures for Statistical Computing in Python. in 56–61 (2010). doi:10.25080/Majora-92bf1922-00a.

29. Hunter, J. D. Matplotlib: A 2D Graphics Environment. *Comput Sci Eng* **9**, 90–95 (2007).

30. The PyMOL Molecular Graphics. *Version 2.0 Schrödinger, LLC.* Preprint at https://pymol.org/2/support.html%0Ahttps://scholar.google.com/scholar?hl=en&as_sdt=0%2C5&q=The+PyMOL+Molecular+Graphics+System%2C+Version+1.74.4+Schrodinger%2C+LLC.+https%3A%2F%2Fpymol.org%2F+%3B+Accessed+10+February+2020.&btnG=%0Ahttps://pymol.org/2/supp.

31. Sehnal, D. *et al.* Mol*Viewer: Modern web app for 3D visualization and analysis of large biomolecular structures. *Nucleic Acids Res* **49**, (2021).

32. Virtanen, P. *et al.* SciPy 1.0: fundamental algorithms for scientific computing in Python. *Nat Methods* **17**, 261–272 (2020).
