# Supplementary material for: Differentiable rotamer sampling with molecular force fields
Source: Brief Bioinform. 2023 Dec 12;25(1):bbad456. doi: 10.1093/bib/bbad456 (PMC10720392; doi:10.1093/bib/bbad456)
Supplement: Supplemental_Tables_bbad456 [file supplemental_tables_bbad456.docx]

Table S1: Units of measurement and specific numerical values used to represent molecules and their properties in OpenMM and PyTorch. This table may be used to convert the numerical outputs of the computations to predicted measurements.

| Physical quantity | Physical units | Numerical value during computation |
| --- | --- | --- |
| distance $(d_{ij})$ | nanometer $(\text{nm})$ | $1$ |
| energy $(E, \epsilon_{ij})$ | kilojoule / mole $(\text{kJ} \text{mol}^{-1})$ | $1$ |
| electric charge $(q)$ | elementary charge $(e= 1.609\times{10}^{-19} \text{C})$ | $1$ |
| Avogadro’s constant $(N_{A})$ | unitless | $6.022\times{10}^{23}$ |
| Ideal gas constant $(R)$ | kilojoule / (mole Kelvin) $(\text{kJ} \text{mol}^{-1} \text{K}^{-1})$ | $8.314\times{10}^{-3}$ |
| Coulomb constant $\left( \frac{1}{4\pi\epsilon_{0}} \right)$ | $\text{kJ} \text{nm} \text{mol}^{-1} e^{-2}$ | $138.9354576$ |
| Solute (protein) relative electric permittivity $(\epsilon_{solute})$ | unitless | $1$ |
| Solvent (water) relative electric permittivity $(\epsilon_{solvent})$ | unitless | $78.5$ |
